# Quantifying Spillover Risk with an Integrated Bat-Rabies Dynamic Modeling Framework

**DOI:** 10.1155/2023/2611577

**Published:** 2023-06-20

**Authors:** Eva Janoušková, Jennifer Rokhsar, Manuel Jara, Mahbod Entezami, Daniel L. Horton, Ricardo Augusto Dias, Gustavo Machado, Joaquín M. Prada

**Affiliations:** ^1^School of Veterinary Medicine, Faculty of Health and Medical Sciences, University of Surrey, Guildford, UK; ^2^Department of Population Health and Pathobiology, College of Veterinary Medicine, North Carolina State University, Raleigh, NC, USA; ^3^Department of Preventive Veterinary Medicine and Animal Health, School of Veterinary Medicine, University of Sao Paulo, Sao Paulo, Brazil

## Abstract

Vampire bat-transmitted rabies has recently become the leading cause of rabies mortality in both humans and livestock in Latin America. Evaluating risk of transmission from bats to other animal species has thus become a priority in the region. An integrated bat-rabies dynamic modeling framework quantifying spillover risk to cattle farms was developed. The model is spatially explicit and is calibrated to the state of São Paulo, using real roost and farm locations. Roost and farm characteristics, as well as environmental data through an ecological niche model, are used to modulate rabies transmission. Interventions aimed at reducing risk in roosts (such as bat culling or vaccination) and in farms (cattle vaccination) were considered as control strategies. Both interventions significantly reduce the number of outbreaks in farms and disease spread (based on distance from source), with control in roosts being a significantly better intervention. High-risk areas were also identified, which can support ongoing programs, leading to more effective control interventions.

## 1. Introduction

Bats have long been associated with highly pathogenic zoonoses affecting domestic animal and human hosts [1]. Despite attempts to understand cross-species pathogen transmission from reservoir hosts to recipient hosts, there are still gaps in knowledge regarding the environmental conditions and mechanisms necessary for spillover events to occur [2].

In Latin America, vampire-bat-driven rabies (VBR) has come to attention as both an underappreciated and growing threat [3] and is now the leading cause of both human and livestock rabies mortality in Latin America [4, 5]. VBR is responsible for substantial agricultural and subsequent monetary losses, disproportionately affecting resource-poor farming communities that depend on agricultural economy [6]. It has been recently estimated that tens of thousands of livestock die of VBR annually, corresponding to financial losses between 30 and 50 million USD in the region [4, 7]. VBR is a member of the *Lyssavirus* genus, and similar to other lyssaviruses, disease pathology is marked by acute fatal encephalitis [8, 9]. Of the three species of hematophagous bats, *Desmodus rotundus* (Chiroptera: Phyllostomidae) is the most abundant and prefers to feed on livestock blood [5, 10]; this preference displays the species ability to adapt to anthropogenic ecological changes as it is believed during the pre-Columbian era *D. rotundus* fed upon the indigenous wildlife [11]. Instead of being negatively impacted by urbanization, deforestation, and a resultant decrease in wild prey, *D. rotundus* adapted to the new food sources resulting in an artificially high population [11, 12]. The population changes might have implications for disease dynamics.

Mathematical modeling has been used extensively to understand spread dynamics and improve surveillance and control strategies for many infectious diseases [13–16]. Several frameworks have been proposed to model the dynamics of rabies transmission, approaching the problem from different perspectives [17–21]. Here we present a stochastic network model designed to capture the spatial heterogeneity of VBR transmission between known bat roosts in the state of São Paulo, Brazil, and spillover events into the local cattle farms. We explore the effect of different combinations of current reactive interventions, namely, vaccination of cattle in confirmed VBR-positive farms and other nearby farms, and vampire bat roost control in surrounding areas [22]. While vaccinating cattle can be logistically easier to implement, as there is no need to find remote roost locations, it only protects animals bitten by bats in those farms and thus has no appreciable effect on rabies spread across the region. Preventive interventions do not happen in São Paulo state, as vaccination is not mandatory since 2008. Only reactive vaccination may happen when farmers had a rabies outbreak in their farm or nearby farm. No coordination of vaccination in high-risk areas is made by the official veterinary service, and vaccination has always been paid by the farmers. Currently, as a roost control, warfarin is applied on the back of the captured vampire bats as anticoagulant paste that is spread between bats by themselves during socializing and grooming, and they subsequently die of hemorrhage [11]. This is hoped to reduce incidence in bats by depleting the susceptible (and potentially infected) bat population; however, both ethical and scientific arguments exist against bat culling [23]. Moreover, indiscriminate culling may lead to social disruptions in the roosts, which facilitates pathogen spread [4, 22]. An arguably more effective alternative in the form of a spreadable vaccine may be administrated in a similar manner to protect the bat population from infection [7, 24, 25]. These vaccines at various stages of development have not yet been widely deployed in wild bat populations.

In this paper, we quantify the effect of these interventions in reducing the number of outbreaks and the spatial spread of rabies infection in the state of São Paulo, Brazil. We also provide risk maps for each combination of current control measures used in the area of interest, the state of São Paulo, Brazil.

## 2. Materials and Methods

### 2.1. Study Area and Databases

**Figure 1:**
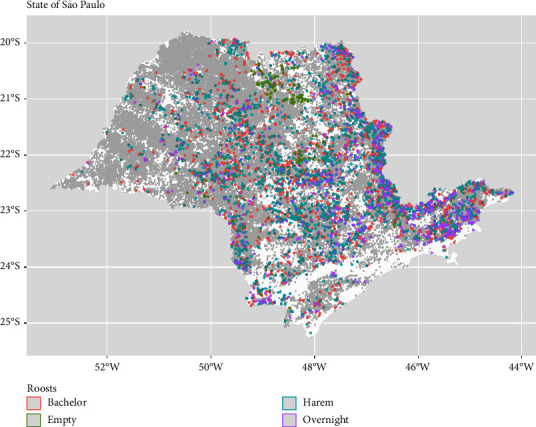
Geographic location of bat roosts visited in 2017-2018, colored by roost type, and cattle farms, gray dots, in the state of São Paulo.

A mathematical modeling framework was developed that broadly represents disease dynamics of VBR transmitted between *D. rotundus* roosts and cattle farms within the state of São Paulo, Brazil. Data on bat and roost ecology have been generated from long-term studies carried out in the state of São Paulo, Brazil, for the past 20 years [11, 22]. The data on roosts and farms used in this study were collated from the more recent surveillance survey carried out in 2017-2018 by the Coordenadoria de Defesa Agropecuária (CDA), the São Paulo State animal health service. The data contain information such as location (municipality, latitude and longitude coordinates, and elevation), information about the farms (number of cattle), and roost specifications (roost types with information about population demographics) on 132,787 farms and 5,170 roosts in São Paulo. The roosts were categorized as either “harems,” if occupied mostly by females and pups; “bachelor,” if dominated by young males; “overnight” if it is only a transit location to rest during foraging and digestion; and “empty” if the location is never occupied by vampire bats [22]. This information from the Coordenadoria de Defesa Agropecuária is then passed to the Ministry of Agriculture and Livestock, from whom cattle farm locations can also be obtained. The farms with no cattle (50,556 farms), as well as empty and overnight roosts (971 roosts), were removed from the dataset, as this study focuses on infection spillover exclusively to cattle and it is believed that the empty and overnight roosts contribute negligibly to rabies transmission. After data cleaning and data quality control checks (i.e., correcting longitude/latitude entry errors where possible, and removing data where it is not possible to correct the entry errors, along with removing duplicated or incomplete records; 6,956 farms and 32 roots removed), our modeling simulations were carried out on 4,167 bat roosts (2,186 bachelors and 1,981 harems) and 75,275 cattle farms (Figure 1).

### 2.2. Model Description

We have developed a stochastic network two-species metapopulation model, linking bat populations (roosts) to cattle populations (farms), through a discrete-time state-based Markov chain model. The state of each population (roost or farm) changes at every discrete daily time step in a probabilistic manner according to a set of rules (see model details in Supporting Information: model description).

**Figure 2:**
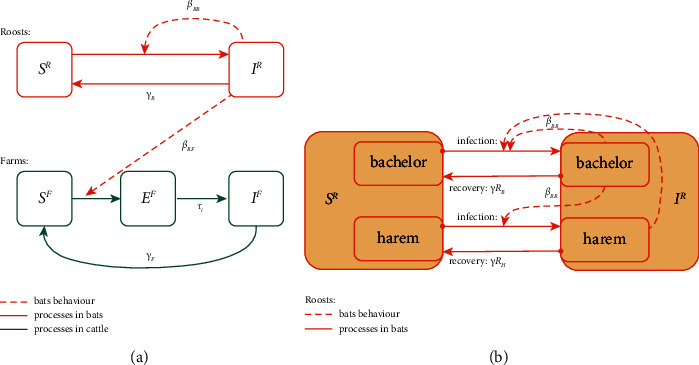
(a) Model schematic for the transmission of bat rabies virus between bat roosts and cattle farms. (b) Detailed between-roost dynamics schematic. The state changes between epidemiological classes are shown by solid arrows. The parameters affecting the state changes are displayed, see also Table 1. Dashed arrows represent virus transmission. No interventions are included in these diagrams.

We consider two possible states for the roosts and three possible states for the farms, Figure 2(a). A roost is defined as susceptible, *S*^*R*^, when rabies is not present and infectious, *I*^*R*^, otherwise, i.e., when there is at least one infectious bat in the roost, and hence the infection spread from the roost is possible. Susceptible roosts become infectious by interacting with an infectious roost and can recover (i.e., become susceptible again) after a period of time (Table 1). Similarly, a farm is susceptible, *S*^*F*^, if there is no infected cattle animal with rabies. Farms where an animal is infected by a bat from an infectious roost become exposed, *E*^*F*^, with infection present, but undetected. The detection time period is drawn from *lognormal* distribution for the farm once its status changed from susceptible to exposed. After this time to detection has past, the infection can be detected in the farm, and thus the farm will be considered infected, *I*^*F*^ (Table 1, Supporting Information: Figure S1). As this parameter can be relatively uncertain, we conducted additional simulations based on more recent estimations (see Supporting Information: distribution of detection period comparison). A farm with a detected infection can recover and become susceptible again (Figure 2(a), Table 1).

Roosts can be composed of young males (i.e., a bachelor roost, *R*_*B*_) or be female dominated (i.e., a harem roost, *R*_*H*_). We assume that the drivers of rabies transmission are the bachelor roosts, such that bachelors can transmit and acquire the infection from other roosts (bachelor or harem), while the harems can only acquire and transmit the infection to bachelors, as male bats are generally the ones traveling between bachelors and harems [30, 31]. The recovery rate differs between bachelor and harem as the longevity of male and female bats differs (Figure 2(b), Table 1). Roost sizes are assumed to be fixed and relatively small (20 individuals in bachelor roosts, 100 in harems), in line with the data collected in the region [11].

The populations, roosts and farms, are connected through a distance-based contact network, assumed to be time-invariant [22]. Only contacts that could lead to disease transmission are considered, such as interactions between two roosts representing males competing for access to females or to roosts with females, i.e., male-driven transmission, or between a roost and a farm representing bats feeding on cattle, expressed by the edges in the network. The transmission is limited up to 10 kilometers flight distance [3]. The bats are expected to feed only in farms at a lower altitude than their roost [11], and thus spillover events are limited by this in the model as well. Contacts between two farms were not considered, transmission usually occurs via a bite or scratch of an infected bat, and consequently, rabies transmission between farms via movement of infected cattle is highly unlikely (Supporting Information: Figure S2).

The risk of rabies virus transmission depends on spatial interactions subjected to a gravity model. The probability of bat movement decreases with longer distance to minimize spent energy; however, it increases with higher number of bats within the roost, harems in our model, as they may fly to further distance due to increased competition, and the roosts with more individuals attract more bats contacts (spatial interaction in Supporting Information). Within-population dynamics are not considered. We assume that the between-roost transmission risk is further modified according to the environmental drivers of location suitability of both roosts: vegetation, elevation, temperature, precipitation, and night time light. These environmental data on roosts were used to calculate suitability indexes by ecological niche model (ENM) (ecological niche model in Supporting Information: Figure S3 and Table S1) [32–34]. The more suitable locations of roosts are expected to attract more bats. The edges in the network are weighted by the risk of rabies virus transmission and between two roosts also by their average environment suitability, where the edges do not exist as described above, i.e., the risk of transmission is negligible, and it is expressed as zero weight of the edge (network weights in Supporting Information). The network weights drive the probability of transmission. The probability of a susceptible population to become infectious or exposed, if it is a roost or a farm, respectively, depends on the sum of the weights of all edges connecting the population with an infectious roost (probability of status changes due to bat behavior in Supporting Information).

We considered two possible rabies interventions in the model: a reactive vaccination of animals in the infected premise and all surrounding farms in a 10 km radius; and/or a reactive roost control in the surrounding area within a 10 km radius from the infected premise (see Supporting Information: Figure S4 for model schematic for the transmission of bat rabies virus between bat roosts and cattle farms including both interventions).

The reactive vaccination of farms is modeled as providing immunity to all farms vaccinated for a year (namely, 365 days). During this time, the vaccinated farms cannot be infected. After a year, the farm loses the immunity, and it will likely be susceptible; however, if the farm was vaccinated while already exposed to infection, but not detected, the farm might be still exposed or infected. If there is a new outbreak in 10 km radius from the vaccinated farm and it is more than a half of year (namely, 182 days) since last vaccination, the animals on the farm are revaccinated.

The roost control is currently based on the administration of a warfarin paste in the back of the captured vampire bats so that during social grooming, conspecifics ingest the paste and indistinctly die of hemorrhage [11, 22]. Such roost control results in the death of nearly all vampire bats in the roost [35], and hence we assume that it leads to an empty roost, which likely will not be repopulated for a long time and will not contribute to the virus transmission until is repopulated. Similarly, if we assume that the spreadable vaccine provides immunity for at least one year, the treated roosts can be assumed to not contribute to transmission [7, 24]. Consequently, for the purposes of modeling the roost control for one year, we assume that if a roost receives an intervention (culling or vaccination), all transmission ceases. To account for a reduced infection pressure when roosts are controlled, we assumed an increased recovery rate for farms when the roost control is carried out (Table 1).

### 2.3. Model Calibration

The model was calibrated to the data available from the region of São Paulo acquired in 2017-2018 when vaccination of farms, but no roost control, was performed in the area. Two model parameters, the roost-to-roost transmission rate, *β*_*RR*_, and the roost-to-farm transmission rate, *β*_*RF*_, were fitted (see below); the remaining parameters were extracted from the literature (Table 1).

Model fitting was carried out using a regression-based conditional density Approximate Bayesian Computation algorithm, such as implemented in Prada Jiménez de Cisneros et al. [36], following Beaumont et al. [37] and Lopes and Beaumont [38]. In brief, summary statistics were calculated from the 2017-2018 data, and we ran a total of 7,800 simulations to calibrate the roost-to-roost and roost-to-farm transmission rates, *β*_*RR*_ and *β*_*RF*_, respectively. In the official data, it was reported an average of 6 outbreaks per 1 million cattle per year. This, based on the farm sizes in the region, can be transformed to number of outbreaks across the farms per year (45.2 outbreaks on average). However, rather than trying to fit to an average number of outbreaks per year, due to the yearly stochastic variation, we decided to fit to the total number of outbreaks expected in farms over a five-year period (226). Due to the high number of nodes in the network, the fitting process was carried out in two phases. First, the roost-to-roost transmission rate parameter was calibrated to reach 1% prevalence across all roosts in the network by simulation of 100 of years of transmission between roosts. As it is very difficult to have a positive direct test in a *Desmodus rotundus* survey (but higher in serological surveys), we assumed that prevalence must be low, here assumed to be 1%. The spillover to farms was not considered in this phase; therefore, no intervention was allowed. Second, the roost-to-farm transmission rate parameter was fitted to generate the 226 outbreaks in farms across a five-year period; as we calibrated the parameters using the data collected in 2017-2018 when only farm vaccination was performed, only this intervention was allowed in the second phase of calibration (see model calibration in Supporting Information: Figure S5 and Table S2, for more details).

### 2.4. Bat-Rabies Control Scenarios

Using the calibrated model, we explored several VBR control scenarios, assessing the effect over the spread of VBR from a single introduction in a randomly selected roost. We considered three initial settings of suitability environments, depending on whether the single initial introduction was in either a high, middle, or low suitability environment (limited to roosts connected to at least five other roosts, to ensure simulations are not initiated in isolated locations). We define these three initial sets of roosts, so that the roosts with the suitability index, calculated through the ENM [32–34], within upper decile (after excluding isolated locations) form a set of roosts in high suitability environment, roosts with the suitability index of ± 5% around the median form the middle, and within bottom decile form the low suitability environment sets of roosts (Supporting Information: Figure S6).

**Figure 3:**
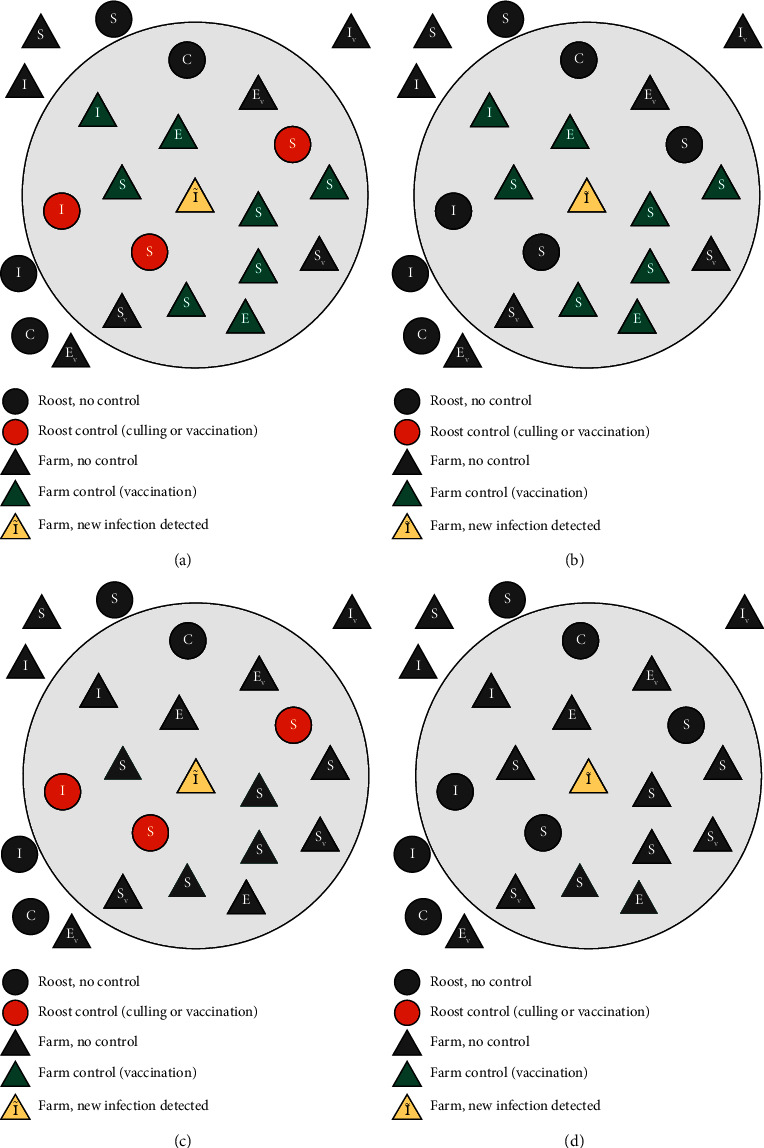
Diagram of the different reactive intervention strategies, summarizing which farm and/or roost will be controlled. An intervention would be implemented in farms and/or roosts within 10 km distance from a detected positive farm (large light gray circle). The only exceptions would be farms recently vaccinated (subindex *V*) that will not be revaccinated again until 6 months have passed since last vaccination and controlled roosts (*C*). Four different intervention strategies were modeled: (a) farm vaccination and roost control, (b) farm vaccination, (c) roost control, and (d) no intervention.

In each initial setting, in response to VBR being detected in farms, we consider all combinations of two reactive interventions included in the model: a combination of roost control and farm vaccination, each intervention alone, or no intervention. Consequently, we simulate 12 different control scenarios (three initial settings of suitability environment with four different intervention strategies, Figure 3). This enables a comparison of the impact of environmental suitability on virus transmission and intervention effectiveness. With the model calibration, we selected the best posterior draws (107 selected), and we ran 50 simulations with each posterior, 5,350 simulations in total per control scenario.

We assessed two different outcomes: (i) the number of detected outbreaks in farms and (ii) the distance of virus spread from initial infection in a roost to a farm in one year, for the different control scenarios. We determined whether there are any statistically significant differences between the means of the outcomes for different intervention strategies by Welch's one-way heteroscedastic *F* test, an alternative to ANOVA robust to the violation of variance homogeneity assumption, which we observed for both outcomes. Welch's test has one of the highest adjusted powers among one-way tests for positively skewed data, which we observed for the numbers of outbreaks, and for approximately normally distributed data, that we observed for the distances [39]. Since we do not confirm the hypotheses of equal means, we perform the Games–Howell post hoc tests to recognize which pairs of intervention strategies significantly differ, assessed through the Holm-corrected *p* values. Tests and visualization are performed using the *ggstatsplot* package of R [40].

Areas at persistently high risk of VBR transmission and spillover in the state of São Paulo after random introductions can be highlighted by mapping spillover events. We divided the state of São Paulo into squares of 3′ latitude times 3′ longitude (30 km^2^). Spillover risk of farms was calculated as a proportion of (detected and undetected) infections among all simulations of a particular scenario.

## 3. Results

**Figure 4:**
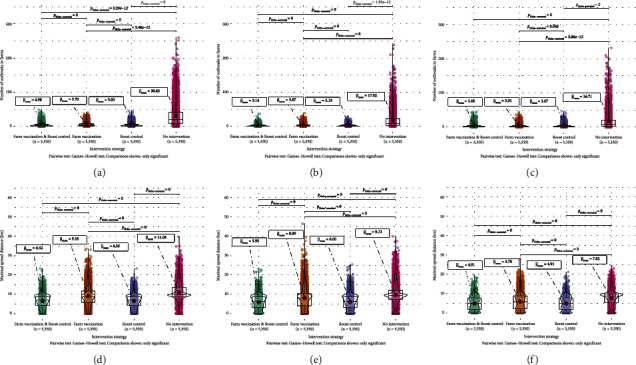
(a–c) Distribution of the number of outbreaks (i.e. infection detections) in farms for different combinations of interventions. (d–f) Distribution of maximal distances of virus spread from a single initial infection in a roost to a farm in one year in kilometers, including no virus spillovers to farms, i.e., zero distances, for different combinations of interventions. The initial suitability environment of a first infected roost is either (a, d) high (90−100th percentile), (b, e) middle (45−55th percentile), or (c, f) low (0–10th percentile). For Welch's *F* test statistics and *p* values for each comparison (a–f) to test the hypothesis of equal means in the four intervention strategies, see Table S3. (a, d) High suitability environment. (b, e) Middle suitability environment. (c, f) Low suitability environment.

The average number of detected outbreaks in farms in a year, from a single introduction, is decreased significantly when an intervention strategy is implemented (being either cattle vaccination, roost control, or both), across all three suitability environments considered (Figures 4(a)–4(c)). As mentioned above, “roost control” represents either bat vaccination or bat culling, both assumed to have perfect efficacy, thus making the roost immune. The maximal distances of virus spread from a single infection in a roost to a farm in one year for the different intervention strategies are shown in Figures 4(d)–4(f).

The *F* statistics and the *p* values of Welch's *F* test are summarized for each comparison in Table S3 in Supporting Information, with all *p* values close to zero, i.e., for both outcomes, across all three initial suitability settings, we do not confirm the hypothesis of equal means in the four intervention strategies. The Games–Howell post hoc tests identify which pairs of intervention strategies significantly differ. The Holm-corrected *p* values indicate that the outcomes for the combination of farm vaccination and roost control, versus roost control alone, are not significantly different, Figures 4(a)–4(f). Additionally, when infection starts in a low suitability environment, the number of outbreaks in farms does not significantly differ between the combination of farm vaccination and roost control, versus farm vaccination alone, Figure 4(c).

**Figure 5:**
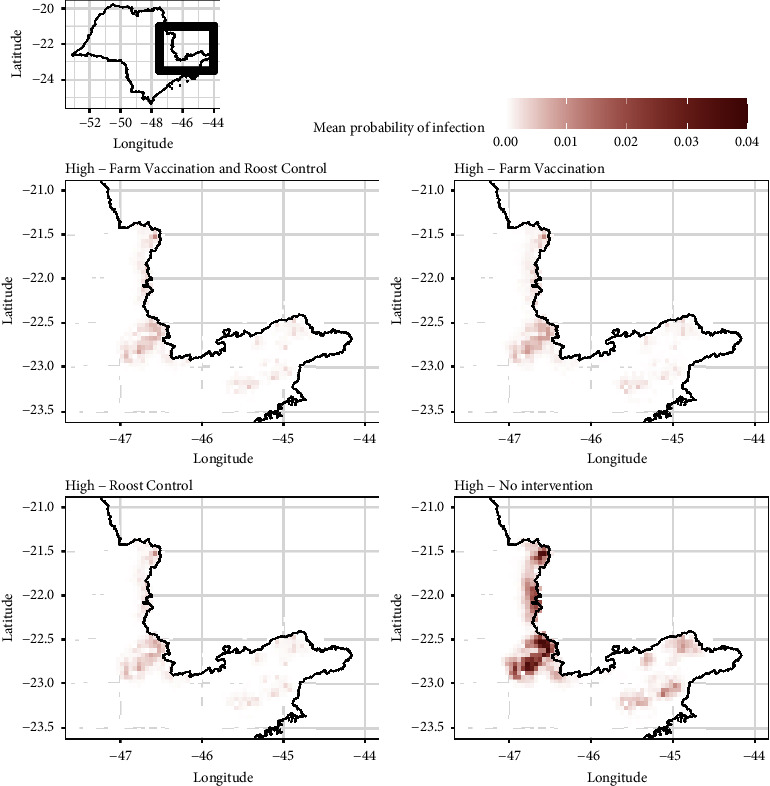
Spillover risk to farms measured as the probability of detected and undetected infections, among all simulations with initial infection in high suitability environment, for each intervention strategy. The value per pixel shown is the average across the farms within the pixel (square 3′ latitude times 3′ longitude, i.e., approx. 30 km^2^).

**Figure 6:**
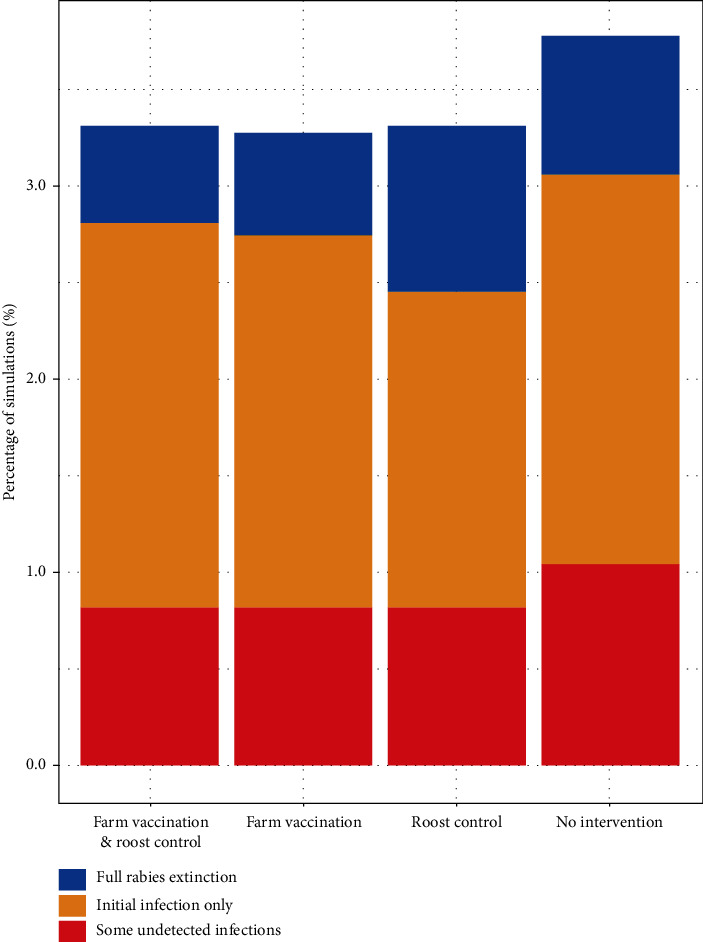
Percentage of runs where rabies is fully extinct (blue), rabies remains only in the initial roost without spreading further (yellow), and rabies had a limited spread to farms but is undetected at the end of the one year simulation period (red), in a high suitability environment for each intervention strategy.

The most ecologically suitable areas for bats, and thus where spread is likely to be higher, are concentrated in the east side of São Paulo state. The infection risk decreases dramatically with any intervention (whether it is farm vaccination, roost control, or both); the probability of an outbreak occurring in farms, after a single introduction, can be as high as 3.81% of the simulations ran without intervention and as low as 1.02% of the simulations with the roost control, Figure 5. Roost control, either standalone or implemented in combination with cattle vaccination, appears to be the most effective intervention strategy, Figures 4 and 5. High infection risk probabilities in farms were also observed in the middle and low suitability environments, which could be as high as 7.12% (Supporting Information: Figures S7 and S8). On the other hand, across all the simulations and all scenarios, there is an approximately 3% chance that rabies has a very limited spread, either by going completely extinct, remaining in the original roost without spreading further or with a limited (undetected) spread in farms, Figure 6.

## 4. Discussion

The aim of this study was to explore the spatiotemporal dynamics of vampire-bat-driven rabies (VBR) in São Paulo, Brazil, and identify high-risk areas of spillover to cattle farms. This was achieved through the development of a novel stochastic network two-species metapopulation model. The model was used to explore the impact of current interventions, ring vaccination of farms, and/or ring roost control (either bat culling or bat vaccination) around a positive farm. Our results suggest that either strategy can prevent substantial number of on-farm outbreaks as well as significantly reduce the geographical spread of these outbreaks (i.e., distance from initially infected roost to the furthest infected farm). However, roost control alone or combined with farm vaccination in general leads to more significant control results than cattle vaccination alone. Interestingly, the combination of both interventions did not provide a significant benefit comparable to roost control alone. We also found areas of consistently high infection risk in high roost suitability environments and in middle and low suitability environments for bat roosting.

As possibly the most diverse, abundant, and geographically dispersed vertebrate, bats are unique in their ability to fly, long lifespans, migratory patterns, and in hosting a diverse suite of pathogens including rabies virus [41, 42]. Some of these factors contribute towards the efficacy of bats as zoonosis transmitters but also towards the lack of data about pathogen circulation, in particular their high level of mobility and vast geographic ranges, as field data are often collected from a subset of a species geographic range over a small timescale [3]. While keeping the model relatively simple in terms of bat demography, we account for several important environmental drivers of disease transmission, such as elevation driving the explicit range of contact between roots and farms, male-driven transmission between bat roosts, flight distance, and environmental suitability. This is key to generate useful risk maps that can support policy implementation. For example, Benavides et al. [4] highlighted the challenge of applying bat vaccines across many roosts, which could be mitigated by focusing efforts on the areas estimated by the model to be at higher risk, which could in addition reduce cross-species exposure while reducing the impact on bat communities.

Bat culling remains a controversial approach to VBR control [43]. Alternatively, a spreadable vaccine may be administrated similar as the vampiricide. Laboratory and model results showed that the oral vaccination could be effective [7, 24]. In the model, we considered the implementation of a roost control, which can either represent bat culling or bat vaccination. Either way, it is modeled so that if a roost receives the intervention, all transmission ceases for at least one year. This is likely on the upper range of operational feasibility, particularly for areas where capture in roosts is impossible and control is done by catching bats at foraging locations. In the case of culling, the spread of the poison due to intensive grooming is intended to lead to an empty roost [35]. However, reports indicate that only a small number of *D. rotundus* are eliminated using warfarin in Brazil [44]. Even if roost would be completely empty, they would likely be repopulated in the future, but this could potentially take a long time and has dangerous ecological implications. In fact, recolonization is an added complexity for such a model, which here can be side-stepped somewhat by measuring dynamics over a short period of time (in our case one year). Some empirical observations from São Paulo suggest the population could recompose as fast as three years after culling, but there is not a lot of data available to narrow this value. Furthermore, it was suggested that culling may increase recruitment of susceptible juveniles into the system, making the intervention ineffective or counterproductive; therefore, the efficacy simulated here is likely overestimated in case of culling [20, 43]. To study this in more detail, the model would need to be modified to include within roost dynamics. However, little is known about the immunological processes related to lyssavirus infection within *D. rotundus*; this knowledge gap includes seroconversion/seroreversion rates and transmissibility [45]. Although modeling may provide insight into the incidence of infection at the roost level, the incidence and therefore prevalence within the roosts remains an uncertainty in our metapopulation model. A vast amount of research has been undertaken and is currently underway pertaining to the immunological complexities that allow many bat species to serve as reservoirs for highly pathogenic viruses and coexist without suffering several pathological consequences [18]. Several theories have been proposed for how this is possible, but an in-depth review of individual bat immunology and immunogenetics is beyond the scope of this paper. Moreover, bat vaccination, being spread the same way, could lead to a high proportion of immunity in the roost population, arguably for at least one year, if the coverage is sufficient, which is challenging to achieve in the field [7, 25]. This type of control would not change the population structure within the roost; on the other hand, it will not reduce the impacts of bats as pests causing harm to animals by bat bites independently of rabies, including skin damage, anemia, loss of vision, loss of weight and productivity, and predisposition to other infection [46].

Nevertheless, cattle vaccination has also achieved considerable reduction in on-farm outbreaks and geographical spread of these outbreaks across the three initial suitability environments. As cattle vaccination cannot in itself reduce transmission between roosts, this counter-intuitive result is likely due to ring vaccination in cattle protecting the farms further away from the source of infection. Either way, a spatial mathematical model simulating the impact of these type of interventions, for example, extending the one presented here, could be used by programs to evaluate the consequences of their introduction and identify the most suitable locations to cover with the campaign for the successfully control, or even eradication, of the virus. As concluded by Blackwood et al. [18], who developed several stochastic SEIR models examining viral persistence, bat population migration, and the effects of bat population culling, the mechanisms to reduce spillover via viral elimination likely need to be spatially coordinated to be effective as we demonstrated here.

In the model, we considered the minimum delay in the detection of outbreaks in the cattle farms to be 25 days, with a mean detection time around 75 days, and the most frequently observed delay (mode) of 30 days. We assumed a relatively over dispersed distribution to capture both the latency period and delay in detection. We also explored an alternative, shorter, detection time, shown in the Supplementary Information: distribution of detection period comparison, with qualitatively similar results (see Figures S9–S11), but a higher impact of the different interventions compared to a no intervention scenario. As the interventions simulated here are reactive, reducing the delay in detection could generate significant gains in reducing transmission. Alternatively, the farm or roost control could be administered in a prospective manner, for example, focusing on high-risk areas. The challenge would be to justify the investment to stakeholders (whether it is the farmers paying for the cattle vaccine, or the government paying for either farm or roost control), when the risk might not be perceived.

We followed prior work made in the region [11, 22], building a similar contact network as in Rocha and Dias [22], with a consistent assumption of up to 10 km flight distance [3] and dependence of bat foraging migration pattern on altitude [11]. How far within the 10 km distance the bats fly is determined by the number of individuals in the roost, since individuals may fly to more distant feeding sources and/or roosts to minimize competition with conspecifics [11, 47]. We address these spatial interactions by utilizing a gravity model. In addition, we incorporate knowledge of favorable conditions for bats using the roost locations and an ecological niche model to capture the environmental suitability (ecological niche model in Supporting Information). Our approach has however a number of limitations; the contact network is assumed to be time-invariant and we are examining outbreaks over a one-year time period from a single introduction. Assuming a unique infected roost at random (potentially in the middle of the region) as a starting point is unrealistic; however, it allows us to better capture the expected spatial spread from a single point. In an endemic situation, such as São Paulo, there will be multiple infected roosts in different locations at the same time; however, modeling an endemic situation would make it challenging to evaluate propagation, as different infected roots may be affecting the same susceptible roosts and farms. The model focuses only on spillover to cattle; however, other animals are in risk (e.g., horses), and since rabies virus is zoonosis, spillover to humans occurs. It is also possible that not all roosts are known; however, it is likely that most of the roots were recorded, since most of São Paulo State is highly anthropized and accessible, and thus if there are unknown (unregistered) roosts, their proportion is insignificant. Moreover, the known roosts are systematically visited (at least yearly), and if empty, the vicinity of affected (by bat bites) farms is revisited by the official veterinary service in search of new roosts.

The reactive interventions depend on reports from the producers which is influenced by many socioecological factors; similarly, adherence to intervention and thus vaccination of the animals when infection nearby is reported might be conditioned by various factors [6]. It is worth noting that we assumed that cattle vaccination and roost elimination were 100% effective, which indeed is rarely the case in VBR control. Further interaction of this model should considered variation of the effectiveness of vaccination, which could include the effects of yearly campaigns, or vaccinating a proportion of cattle based on VBR within herd incidence. The behavior effects on intervention need to be accounted for in the model if we want more realistic predictions. Furthermore, the intervention strategies effective to reach programmatic goals need to be evaluated in economic manner as the government and farmers' financial sources are limited [48]. For example, anemia from bat bites may reduce livestock productivity [7], hence making a difference in bat culling compared to bat vaccination. Roost control might be more cost-efficient to the official service since a smaller number of locations should be visited and vaccine delivery (for example as a paste) is more straightforward than cattle vaccination. The model presented here does not evaluate the economic implications and therefore distinguishes only between susceptible, exposed, and infected farms, ignoring how many animals are present and to which extent they are affected by bites and/or infection. The cumulative losses due to bites if no culling is performed and even deaths if no roost control is in place might markedly change the cost-effectiveness. Last, but not least, the trust, support, and commitment of stakeholders and involved institutions are necessary to reach the expected results [48]. For instance, vaccination of vampire bats without population reduction will be unacceptable to some stakeholders since uncontrolled bat depredation sustains exposures to non-rabies pathogens [7]. The stakeholder's preferences have to be taken into account when assessing the sustainability of the interventions.

## 5. Conclusion

We have developed a novel stochastic network two-species metapopulation model that captures transmission of VBR between bat roosts as well as spillover events to cattle farms. After exploring two alternative control strategies, namely, reactive ring roost control (i.e., bat culling or bat vaccination) and reactive ring cattle farm vaccination, we found no large differences in their expected efficacy; however, interventions in roosts were statistically significantly better in all settings considered across both outcomes (number of outbreaks and spatial spread from initial introduction). Such mathematical frameworks can prove useful to inform control interventions, particularly identifying high-risk areas where prospective vaccination, either in cattle or in bats, could take place. This will support ongoing programs, leading to more effective control. Nonetheless, to reach long-term strategies and sustainability that could move beyond control to potential local elimination and eradication, human behavior, for example, in context of interventions uptake and response to VBR infection in farms, needs to be incorporated in model to get more accurate predictions. In addition to assessing intervention strategies' effectiveness and high-risk areas such as provided in this study, economic evaluation is essential before decision is made on interventions.

## Figures and Tables

**Table 1:** Summary of model parameters.

Parameter	Value	References
Roost recovery rate (days^−1^), *γ*_*R*_*H*__ and *γ*_*R*_*B*__	1.71 *∗* 10^−4^ and 1.61 *∗* 10^−4^	[26]
Detection time in farm (days), *τ*_*i*_	∼lognormal (*μ* = 3.14, *σ* = 1.24), shifted by 25	[27–29]
Farm recovery rate (days^−1^), *γ*_*F*_	16.44 *∗* 10^−3^ (if roost control performed)	Empirical observation by Dr Dias, from experience in São Paulo, such as in [11, 22]
	5.48 *∗* 10^−3^ (if roost control not performed)	
Roost-to-roost transmission rate, *β*_*RR*_	8.99 *∗* 10^−2^–13.36^1^; see Figure S5 in SI^2^ for posterior distribution	Calibrated
Roost-to-farm transmission rate, *β*_*RF*_	154.37–693.80^1^; see Figure S5 in SI^2^ for posterior distribution	Calibrated

^1^Calibrated with distances in meters. ^2^SI stands for Supporting Information.

## Data Availability

Some of the data that support the findings of this study are not publicly available as they are protected by confidential agreements. All code generated is available on GitHub: https://github.com/machado-lab/Quantifying-spillover-risk-with-an-integrated-bat-rabies-dynamics.

## Supplementary Materials

Supplementary Materials Figure S1: distributions assumed for the two detection time periods in farms. Figure S2: network illustration from a view of a harem roost in the middle. The gray area represents the distance of 10 km from the roost in the middle. The directed edges represent the possibility of VBR transmission from or to the harem (the edges are directed from a potential infection donor to a potential infection recipient). Figure S3: suitability map of VBR in the state of São Paulo. Figure S4: model schematic for the transmission of bat rabies virus between bat roosts and cattle farms including both interventions (roost control and farm vaccination). The state changes between epidemiological classes are shown by solid arrows. The parameters affecting the state changes are displayed, see also Table 1. Dashed arrows represent virus transmission. Dotted arrows represent removal of the population. Figure S5: posterior distribution of the roost-to-roost transmission rate, *β*_RR_, and the roost-to-farm transmission rate, *β*_RF_. Calibrated with (A) DD1; (B) DD2. Figure S6: settings for three initial scenarios. For each initial scenario, a set of roosts is defined, from which one roost is randomly selected for each simulation as the first introduction of infection. Three levels of environmental suitability are defined as high, 90–100th percentile (between the roosts connected to at least five other roosts), middle, 45–55th percentile, and low, 0–10th percentile of suitability indexes. Figure S7: spillover risk to farms measured as the probability of detected and undetected infections, among all simulations with initial infection in middle suitability environment, for each intervention strategy. The value per pixel shown is the average across the farms within the pixel (3′ latitude times 3′ longitude, i.e., approx. 5.5 times 5.5 km square). Figure S8: spillover risk to farms measured as the probability of detected and undetected infections, among all simulations with initial infection in low suitability environment, for each intervention strategy. The value per pixel shown is the average across the farms within the pixel (3′ latitude times 3′ longitude, i.e. approx. 5.5 times 5.5 km square). Figure S9: (A, B) distribution of the number of outbreaks (i.e., infection detections) in farms for different combinations of interventions. (C, D) Distribution of maximal distances of virus spread from a single initial infection in a roost to a farm in one year in kilometers, including no virus spillovers to farms, i.e., zero distances, for different combinations of interventions in a high suitability setting. Figure S10: spillover risk to farms measured as the probability of detected and undetected infections, among all simulations with initial infection in high suitability environment, for each intervention strategy. The value per pixel shown is the average across the farms within the pixel (square 3′ latitude times 3′ longitude, i.e., approx. 30 km^2^). This figure is akin to Figure 5 in the main text, here with DD2 instead of DD1. Figure S11: percentage of runs where rabies is fully extinct (blue), rabies remains only in the initial roost without spreading further (yellow), and rabies had a limited spread to farms but is undetected at the end of the one-year simulation period (red), in a high suitability environment for each intervention strategy. This figure is akin to Figure 6 in the main text, here with DD2 instead of DD1. Table S1: list of biotic and abiotic variables used in the ENM. Table S2: details on the two calibration phases to fit the roost-to-roost and roost-to-farm transmission rates, *β*_RR_ and *β*_RF_, for the model. Table S3: results of Welch's one-way heteroscedastic *F* tests for the hypotheses of equal means among different interventions, for two outcomes and three initial settings. Subsequent pairwise test results are displayed in Figure 4.
