# Supplementary material for: Quantifying Spillover Risk with an Integrated Bat-Rabies Dynamic Modeling Framework
Source: Transbound Emerg Dis. 2023 Jun 20;2023:2611577. doi: 10.1155/2023/2611577 (PMC12017007; doi:10.1155/2023/2611577)
Supplement: Supplementary Materials — Figure S1: distributions assumed for the two detection time periods in farms. Figure S2: network illustration from a view of a harem roost in the middle. The gray area represents the distance of 10 km from the roost in the middle. The directed edges represent the possibility of VBR transmission from or to the harem (the edges are directed from a potential infection donor to a potential infection recipient). Figure S3: suitability map of VBR in the state of São Paulo. Figure S4: model schematic for the transmission of bat rabies virus between bat roosts and cattle farms including both interventions (roost control and farm vaccination). The state changes between epidemiological classes are shown by solid arrows. The parameters affecting the state changes are displayed, see also Table 1. Dashed arrows represent virus transmission. Dotted arrows represent removal of the population. Figure S5: posterior distribution of the roost-to-roost transmission rate, βRR, and the roost-to-farm transmission rate, βRF. Calibrated with (A) DD1; (B) DD2. Figure S6: settings for three initial scenarios. For each initial scenario, a set of roosts is defined, from which one roost is randomly selected for each simulation as the first introduction of infection. Three levels of environmental suitability are defined as high, 90–100th percentile (between the roosts connected to at least five other roosts), middle, 45–55th percentile, and low, 0–10th percentile of suitability indexes. Figure S7: spillover risk to farms measured as the probability of detected and undetected infections, among all simulations with initial infection in middle suitability environment, for each intervention strategy. The value per pixel shown is the average across the farms within the pixel (3′ latitude times 3′ longitude, i.e., approx. 5.5 times 5.5 km square). Figure S8: spillover risk to farms measured as the probability of detected and undetected infections, among all simulations with initial infection in low sui [file 2611577.f1.pdf]

Supporting Information

Quantifying spillover risk with an integrated rabies dynamic modeling framework

Eva Janoušková [1], Jennifer Rokhsar [1], Manuel Jara [2], Mahbod Entezami [1], Daniel L. Horton [1], Ricardo Augusto Dias [3], Gustavo Machado [2,*], Joaquín M. Prada [1,*]

[1] School of Veterinary Medicine, Faculty of Health and Medical Sciences, University of Surrey, Guildford, UK

[2] Department of Population Health and Pathobiology, College of Veterinary Medicine, North Carolina State University, Raleigh, NC, USA

[3] Department of Preventive Veterinary Medicine and Animal Health, School of Veterinary Medicine, University of Sao Paulo, Sao Paulo, Brazil

* joint senior authors

Contents

Model description **3**

Detection period in farms 3
Recovery rate 6
Network 7
Spatial interaction 9
Ecological niche model 11
Network weights 15
Probability of status changes due to bats behavior 16
Interventions 17

Model calibration **18**

Outcomes across environmental settings **23**

Distribution of detection period comparison **27**

References **31**

Model description

The mathematical model was developed to broadly represent disease dynamics of VBR transmitted between *Desmodus rotundus* roosts and cattle farms within the state of São Paulo, Brazil. Supplementing the main text, below we detail some of the specific model features.

Detection period in farms

The detection time (period) in a farm *Fi*, τ_i, is approximated by the incubation period of the first infected cattle animal on the farm, which we assume to follow a *lognormal* distribution reported to be a suitable distribution for incubation periods of infections (Sartwell, 1966; Nishiura, 2007). This distribution was also confirmed as the best fitting distribution for rabies virus incubation period in dogs and cats (Tojinbara et al., 2016). As this period varies with host species (Garg, 2014), we used this distribution but amended the parameters. After the detection time period, the farm-state changes from exposure to infection.

The parameters are unknown and different references may point to a different shape of the distribution. Therefore, we use two different *lognormal* distributions (Figure S1) to evaluate the impact of the detection time distribution. Parameters for the first distribution, as used in the main text and denoted as DD1 in this supplementary, include the mode of the incubation period as 30 days, as it is the most commonly observed incubation period in cattle (personal communication). The mean is assumed to be 75 days, as incubation period in cattle was estimated to average 2 to 3 months and range from 25 to 152 days (Gylys et al., 1998). Consequently, the detection time period, τ_i, for each exposed farm *Fi* is drawn from a shifted *lognormal* distribution (τ_i ~ *lognormal*(ψ, μ, σ); or *ln*(τ_i - ψ) ~ *N*(μ, σ)), with μ =

3.14449, $\sigma = 1.23897$ and shift $\psi = 25$. The probability that the simulated detection time period is less than 25 is 0, and longer than 152 days is approximately 0.085, thus values outside the range stated by Gylys et al. (1998) are very rare for DD1. These values are used throughout unless explicitly stated otherwise. For the second distribution, denoted as DD2, we use the parameters estimated from experimental data more recently (Mollentze et al., 2020), $\mu = 2.8666, \sigma = 0.1797$ and no shift $\psi = 0$. The mode is 17.01862 and mean 17.86326 for this distribution. See distribution of detection period comparison section for the results using this distribution in a high suitability setting.

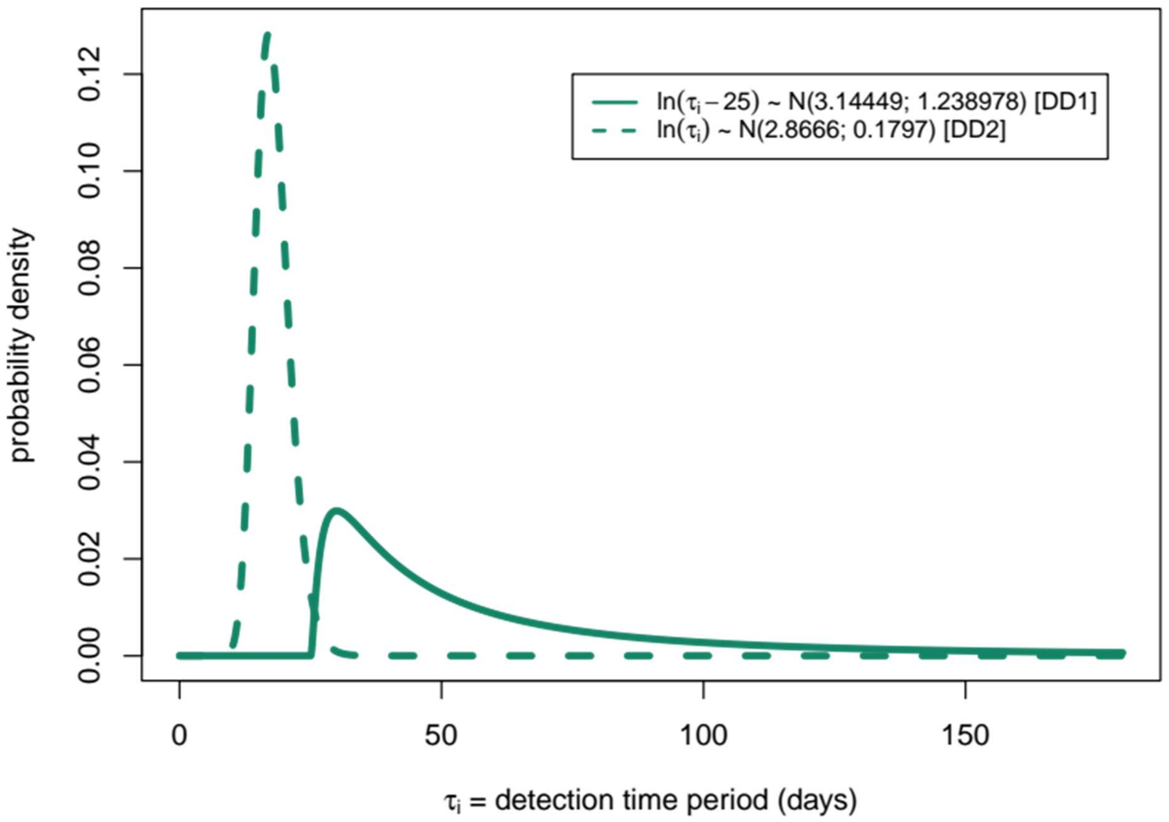

Figure S1 Distributions assumed for the two detection time period in farms.

Recovery rate

In every step (corresponding to 1 day), each infected (and infectious) roost and each farm with a detected infection may recover with the following probability

$$P(\text{recovery of } I^X) = 1 - exp(-\gamma_X), \text{ where } X \in \{R_H, R_B, F\}. \quad \text{(S1)}$$

A roost is assumed to recover when no more infected bats are present, which occurs when all infected bats die. We expect that, on average, an infectious roost may recover after 16 years or 17 years, if it is harem or bachelor, respectively (approximated from the maximal longevity of female and male bats; Delpietro et al., 2017). Following this, the roost recovery rate is 1/(16*365) $days^{-1}$ for harem (γ_{R_H}) and 1/(17*365) $days^{-1}$ for bachelor (γ_{R_B}) roosts. A farm is assumed to recover when all infected animals on the farm die. To account for a reduced infection pressure when roosts are controlled, the recovery rate of farms (γ_F) is lower, *1/(365/6)* $days^{-1}$, when roosts are controlled, whereas it is *1/(365/2)* $days^{-1}$ when the roost control is not performed, corresponding to 2 and 6 months, respectively (Table 1).

Network

The nodes of the network are the occupied roosts (i.e. bachelors and harems) and farms raising cattle. The empty and overnight roosts as well as farms with no cattle were not included in the network as they do contribute negligibly or not at all to the virus transmission. The connections between roosts, the edges, representing possible virus transmission are bi-directional and symmetrical, since risk contact is independent of which roost donates and which roost receives the infection. However, as male bats are generally the ones traveling between bachelors and harems (Streicker et al., 2016; Becker et al., 2020), transmission between two harem roosts is not considered. On the other hand, the connections between a roost and a farm are directed from the former to the latter, as rabies is transmitted from bats to cattle through bites while feeding. There are no loops (edges connecting a node to itself). It is assumed that bats always fly to a lower elevation when foraging, therefore, the transmission from a roost to a farm is possible only when the roost is located at a higher elevation than the farm (Rocha et al., 2020). The estimated maximal flight distance of bats is *10* km (Benavides at al., 2016), hence the transmission from a roost to surrounding farms in the model is expected to be limited to *10* km distance from the infectious roost (Figure S2).

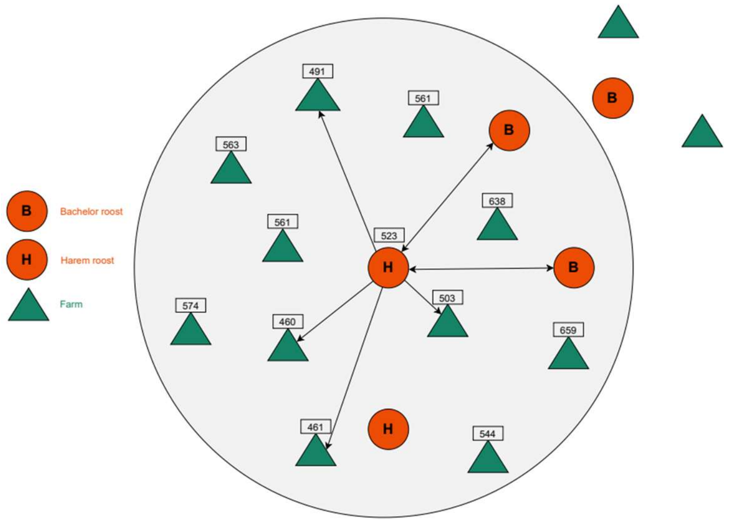

Figure S2 Network illustration from a view of a harem roost in the middle. The gray area represents the distance of *10* km from the roost in the middle. The directed edges represent the possibility of VBR transmission from or to the harem (the edges are directed from a potential infection donor to a potential infection recipient).

Spatial interaction

We used the most common approach to model spatial interactions, a gravity model (Barrios et al., 2012; Maher et al., 2012; Nicolas et al., 2018; Colombi et al., 2020). Using this approach, we estimated the risk of rabies virus transmission between the populations Ri and Xj:

$$r_{Ri,Xj} = \begin{cases} \beta_{RX} * a_{Ri} * a_{Xj} \; / \; \delta^2_{Ri,Xj}, & \text{where } X \in \{R, F\}; \\ 0 & \text{else.} \end{cases} \quad \text{(S2)}$$

The constant β_{RX} is the transmission rate of the rabies virus (β_{RR} or β_{RF} if the population receiving the infection is a roost or a farm, respectively). The transmission rates (β_{RR}, β_{RF}) are fitted to the field data from the state of São Paulo obtained from roost surveillance carried out in the state of São Paulo, Brazil, in 2017-2018 by CDA staff. The Euclidean distances, $d_{Ri,Xj}$, between nodes (as points in the map, disregarding the elevations) were calculated from the latitude and longitude coordinates of the locations using the *distm()* function of the *geosphere* package of R (Hijmans, 2021). How far within the *10* km flight distance the bats actually fly is determined by the number of individuals in the roost, since individuals may fly to more distant feeding sources and/or roosts to minimize competition with conspecifics (Kunz and Fenton, 2003; Rocha et al., 2020). This is captured by the population sizes included in the gravity model. Vampire bats usually form colonies of 20-100 individuals in the state of São Paulo (Rocha et al., 2020). Bachelor roosts were reported by Rocha and Dias (2020) as five times smaller than harems, we thus assume in the model that harems hold 100 bats ($a_{R_H} = 100$), while bachelors have 20 animals ($a_{R_B} = 20$). It is

believed that the number of cattle animals present in a farm does not to influence the number of cattle animals bitten by bats, therefore the numbers of cattle in the farms are not considered (i.e. , $a_F = 1$). The denominator in the gravity model, $\delta_{Ri,Xj}$, takes into account the elevations of both locations (e_{Ri}, e_{Xj}) and is calculated using the Euclidean distances $d_{Ri,Xj}$:

$$\delta_{Ri,Xj} = sqrt(d^2_{Ri,Xj} + (e_{Ri} - e_{Xj})^2). \quad \text{(S3)}$$

Ecological niche model

The environmental suitability of bat roost locations, and whether it is a permanent or temporary shelter, can be explained by the effect of biotic factors, such as vegetation (Escobar et al., 2015), here evaluated using the Enhanced Vegetation Index. Likewise, several studies have also highlighted the importance of abiotic conditions (Hayes and Piaggio, 2018; Lee et al., 2012; Escobar et al., 2015), such as elevation (Giménez et al., 2015), temperature (e.g., temperature seasonality, mean temperature of the coldest month) (Lee et al., 2012), precipitation (Lee et al., 2012) (e.g., precipitation seasonality, precipitation of the wettest and driest month), as well as the impact of nighttime light (Escobar et al., 2015). We developed an ecological niche model (ENM) accounting for those environmental variables, see Table S1, at approximately five kilometers of spatial resolution at the equator. To prevent multicollinearity between the environmental variables, we used VIF (Variance Inflation Factors) implemented in the *sdm* package in R (Naimi and Araújo, 2016). Using this approach, we excluded all the highly correlated variables from the model (VIF value greater than 7), which is associated with a signal of a collinearity problem (Chatterjee and Hadi, 2006).

Table S1 List of biotic and abiotic variables used in the ENM.

Variable	Unit	Source
Temperature seasonality	°C	www.worldclim.org
Mean temperature of the coldest month	°C	www.worldclim.org
Precipitation seasonality	mm	www.worldclim.org
Precipitation of wettest month	mm	www.worldclim.org
Precipitation of the driest month	mm	www.worldclim.org
Elevation	m	www.worldclim.org
Enhanced Vegetation Index (EVI)	EVI index	https://clim-engine.appspot.com/climate Engine
Night time light	Radiance	https://viirsland.gsfc.nasa.gov/

The ENM was developed considering the above-mentioned variables to identify which locations have more favorable conditions for bats, using the roost locations which have been

documented in use or used in the past (Hahn et al., 2014; Razgour et al., 2019; Zeale and Carr, 2019). The study area was determined based on the hypothesis of the most accessible area as the M (see M dimension in the BAM framework proposed by Soberón and Peterson, 2005). To define the study area extent for model calibration, we followed the framework proposed by Soberón and Peterson (2005), which restricts the ENM to ecological features for the organism in question, the resolution of the environmental variables employed, and the extent of the region where the organisms are able to disperse, by their biogeographic barriers. This delimitation of the study area permits to determine the spread potential of the bat populations studied in the geographical area.

To define the M area, we used a buffer zone from the average distance among all roost locations from the further point into each location border of the state of São Paulo, (Figure S3). The total occurrences for each set were randomly subdivided into 70% of the data set for model calibration and 30% for model evaluation. This ENM output was followed as a suitable index for the presence of vampire bat *Desmodus rotundus* (Chiroptera: Phyllostomidae) roost. The suitability indexes, $s_{Ri,}$ calculated using the ENM were then re-scaled between 0 and 1.

The ENM was developed using a presence-background technique that estimates environmental suitability via an index of similarity that resembles a heterogeneous occurrence process or logistic regression function (Phillips et al., 2006). We used Maxent software v3.4.1 (Phillips and Dudík, 2008) with clamping and extrapolation turned off (i.e., no prediction outside the range of environmental conditions in the calibration data) (Anderson, 2013; Owens et al., 2013).

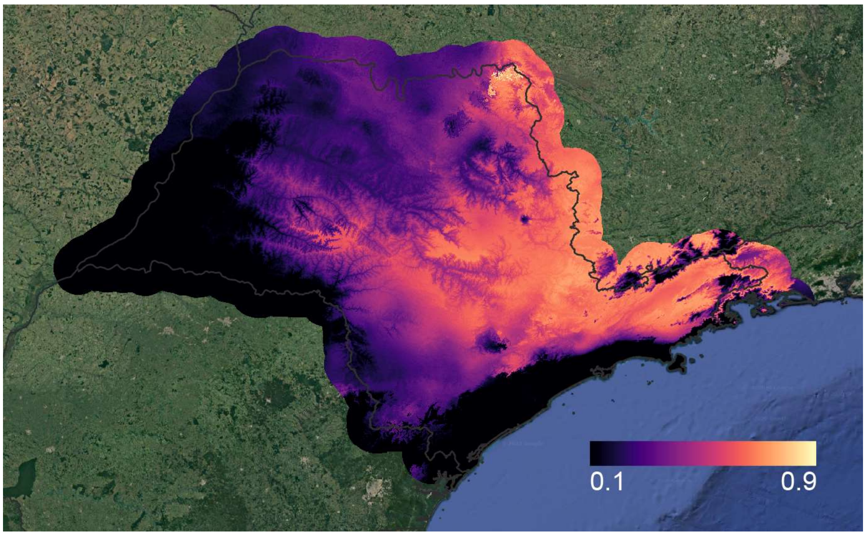

Figure S3 Suitability map of VBR in the state of São Paulo.

Network weights

The edges within the network, representing possible virus transmission, are weighted by the risk of transmission. The calculation of the edge weight, $\omega_{Ri,Xj}$, differ if the edge links two roost nodes or a roost node with a farm node. The risk of transmission for the former is affected by the average of the suitability of the environment of both roosts involved in transmission (s_{Ri}, s_{Rj}) calculated using the ecological niche model (ENM), see *Ecological niche model* section above. Whenever no transmission is possible (e.g. transmission between two harem roosts, transmission to a roost or farm in distance longer than *10* km, or transmission from a roost to a farm in higher elevation as explained in *Network* section above), the weight is zero .

$$\omega_{Ri,Rj} = \begin{cases} r_{Ri,Rj} * (s_{Ri} + s_{Rj}) / 2 & \text{if } i \neq j,\ d_{Ri,Rj} \leq 10\ km, \text{ and } R_i \text{ and } R_j \text{ are not both harems;} \\ 0 & \text{else.} \end{cases} \quad \text{(S4)}$$

$$\omega_{Ri,Fj} = \begin{cases} r_{Ri,Fj} & \text{if } d_{Ri,Fj} \leq 10\ km \text{ and } e_{Ri} > e_{Fj}; \\ 0 & \text{else.} \end{cases} \quad \text{(S5)}$$

Probability of status changes due to bats behavior

The transmission dynamics of the rabies virus between bat populations, roosts (R_i), with a potential to spillover into cattle populations, farms (F_i), are modeled using a discrete-time simulation-based model. At the start of simulation, only one roost is infected, the rest of the roosts and all farms are susceptible. The transition of population j from susceptible to infectious in roosts or to exposed in farms is given by a stochastic risk with the following probability:

$$P(\text{transmission to the population } X_j) = 1 - exp(-\alpha_j), \qquad \text{(S6)}$$

$$\text{where } \alpha_j = \textstyle\sum_{i:R_i \in I^R} \omega_{R_i,X_j}, \text{ and } X \in \{R, F\}.$$

The weights of the edges in the network between the populations R_i and X_j, ω_{R_i,X_j}, express the relative degree of risk of infection transmission from the population R_i to the population X_j (see *Network weights* section above).

Interventions

Two reactive interventions, roost control (culling or vaccination) and farm vaccination, are included in the model (Figure S4), they may or may not be used for a simulation as required. It makes possible to explore impacts of all their combinations, i.e. four different intervention strategies (both intervention, each alone, or neither of them).

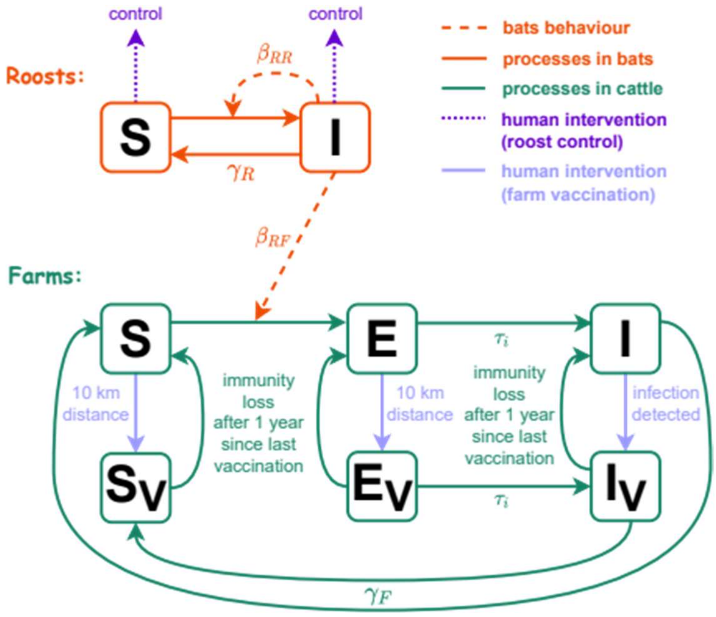

Figure S4 Model schematic for the transmission of bat rabies virus between bat roosts and cattle farms including both interventions (roost control and farm vaccination). The state changes between epidemiological classes are shown by solid arrows. The parameters affecting the state changes are displayed, see also Table 1. Dashed arrows represent virus transmission. Dots arrows represent removal of the population.

Model calibration

A regression-based conditional density Approximate Bayesian Computation (ABC) algorithm (Beaumont et al., 2002; Lopes and Beaumont, 2010; Prada et al., 2014) is used to calibrate the model in two separate calibration phases, fitting the parameters of the vector Θ = (β_{RR}, β_{RF}) one by one. Both calibration phases are described in Table S2. The ABC algorithm can be written as follows:

(1) Specify the model to be calibrated and a posterior setting if applicable.

(2) Specify the unknown parameter θ that will be sampled, and the vector of parameters to be fitted Θ.

(3) Choose a summary statistic S and determine its value s for the observed data.

(4) Choose a tolerance ε.

(5) Sample N times the unknown parameter from the prior distribution, the uniform distribution with a range given in Table S2, $\theta_i \sim p(\theta)$, $i \in \{1, \ldots, N\}$.

(6) Run the model simulation for each sample to get s_i', the value of S for the simulation with $\theta = \theta_i$.

(7) Accept the simulations where $\|s_i' - s\| \leq \varepsilon$, reject other simulations.

(8) Repeat steps 5 to 7 until at least k simulations are accepted.

(9) Calculate the distances of s_i' to s for all accepted simulations as the absolute value of z-score.

(10) Improve the vector of fitted parameters Θ using the weighted linear regression model of S with the vector Θ as the regression coefficients, weighted by the distances calculated in step 9 to weaken the effect of that discrepancy. Keep improved vectors > 0.

(11) These improved vectors Θ obtained in step 10 are an approximation to the posterior distribution $p(\Theta|s)$.

Table S2 Details on the two calibration phases to fit the roost-to-roost and roost-to-farm transmission rates, β_{RR} and β_{RF}, for the model.

	**1st phase of calibration**	**2nd phase of calibration**
Model specification	100 years of transmission between roosts without spillover to farms, therefore no interventions are modeled;<br>starting with one random infection in a roost (limited to those connected to at least 5 other roosts, to ensure	5 years of roost-to-roost as well as roost-to-farm transmission, using a β_{RR}posterior (simulate the model for all posteriors from the 1st phase of calibration), reactive farm vaccination modeled[1];

	simulations are not initiated in isolated roosts)	starting with the posterior equilibrium of infected roosts connected with the posterior rate β_{RR}[4]
Unknown parameter to be sampled, θ	β_{RR}	β_{RF}
Vector of fitted parameters, Θ	(β_{RR})	$\left(\beta_{RR,}\beta_{RF}\right)$
Summary statistics, S	the equilibrium (expected to be reached after 100 years) of infections among roosts (i.e., the infection prevalence among roosts)	the number of expected on-farms outbreaks during simulated 5 years (i.e., the infection incidence on farms)
Observed value of S, s	1% *(for the observed data used in this study: 41.7 roosts)*	an average of 6 outbreaks per 1 million of cattle animals in farms per year *(for the observed data used in this study: 226 on-farms outbreaks in 5 years)*
Acceptance tolerance, ε	+/- 5 infectious roosts	+/- 5 outbreaks in farms

Number of samples for each iteration, N	500	5 (coupled with each β_{RR} accepted in the 1st phase)
Prior distribution[2], $p(\theta)$	$unif(0.1; 15)$	$unif(150; 700)$
Required minimum number of simulations to be accepted, k	100	100
Total number of simulations performed	4,500	3,300[3] with DD1, 4,400[3] with DD2
Number of improved vectors of fitted parameters Θ included in posterior distribution, $p(\Theta\|s)$	110[4]	107 with DD1, 103 with DD2

Notes: [1]At the time of data collection, from which we calculate the observed value of the summary statistics, only reactive farm vaccination was implemented, but no roost control. [2]The prior intervals were determined by pre-simulations on a subset of roosts and farms. [3]The total number of simulations is the number of simulations performed per posterior setting multiplied by the number of posteriors. [4]An equilibrium of infected roosts (roosts infected at the end of the particular simulation of 1st phase of calibration) is attached to each posterior to be used as the posterior equilibrium in the 2nd phase of calibration.

As result of the model calibration, we received posterior distribution of the vector of transmission rates, $\Theta = (\beta_{RR}, \beta_{RF})$ (Figure S5).

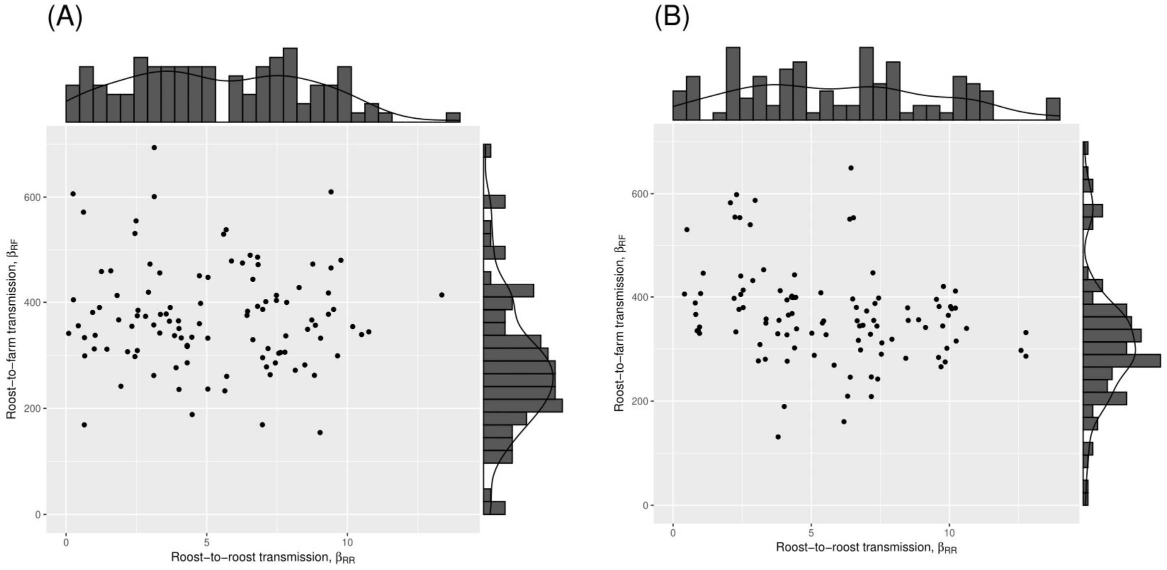

Figure S5 Posterior distribution of the roost-to-roost transmission rate, β_{RR}, and the roost-to-farm transmission rate, β_{RF}. Calibrated with **(A)** DD1; **(B)** DD2.

Outcomes across environmental settings

The results of the Welch's one-way heteroscedastic F tests are summarized in Table S3. This provides the evaluation of the two outcomes, number of outbreaks in farms and maximal spread of disease from origin (i.e. initial infected roost) across the three initial settings for environmental suitability (Figure S6).

The cattle farm locations with higher risk of spillover are detected across the initial environmental settings (Figure 5, and Figure S7).

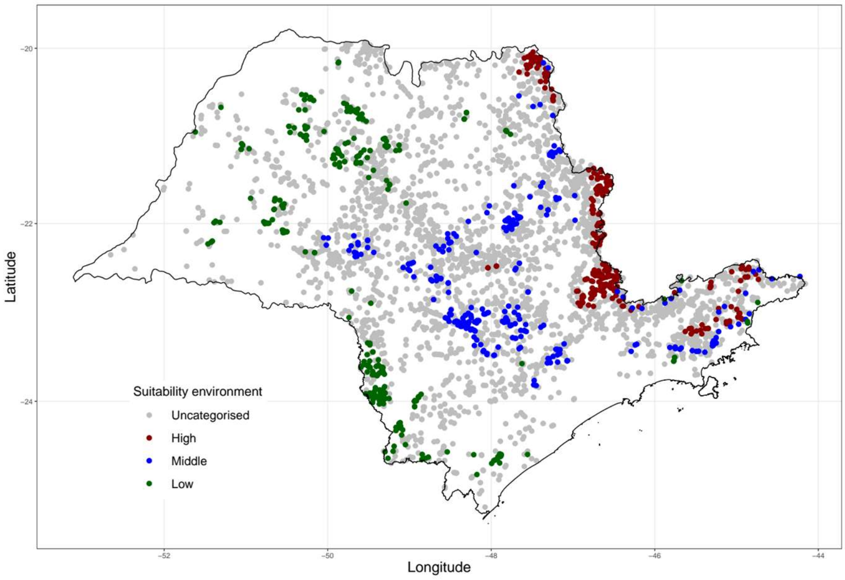

Figure S6 Settings for three initial scenarios. For each initial scenario, a set of roosts is defined, from which one roost is randomly selected for each simulation as the first introduction of infection. Three levels of environmental suitability are defined as high, 90-100th percentile (between the roosts connected to at least five other roosts), middle, 45-55th percentile, and low, 0-10th percentile of suitability indexes.

Table S3 Results of the Welch's one-way heteroscedastic *F* tests for the hypotheses of equal means among different interventions, for two outcomes and three initial settings. Subsequent pairwise test results are displayed in Figure 4.

**Outcome**	**Initial setting (environment suitability)**	***F*(df1, df2)**	**degrees of freedom df1, df2**	***p*-value**
Number of outbreaks in farms	High	1e+03	3, 11482.23	0e+00
	Middle	716.37	3, 11431.00	0e+00
	Low	510.95	3, 11493.78	1.03e−311
Maximal spread distance (km)	High	1.5e+03	3, 11664.57	0e+00
	Middle	805.00	3 ,11689.04	0e+00
	Low	584.50	3, 11835.35	0e+00

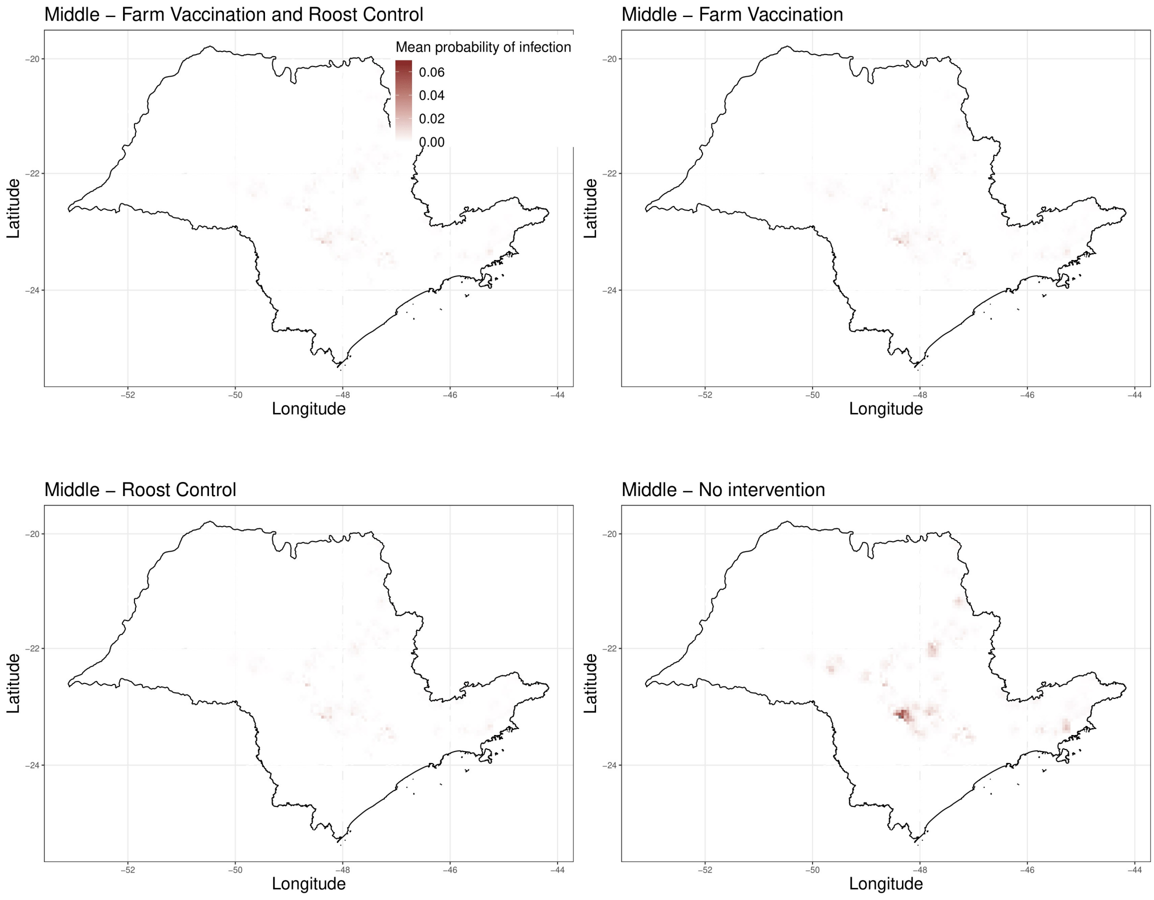

Figure S7 Spillover risk to farms measured as the probability of detected and undetected infections, among all simulations with initial infection in middle suitability environment, for each intervention strategy. The value per pixel shown is the average across the farms within the pixel (3' latitude times 3' longitude, i.e. approx. 5.5 times 5.5 km square).

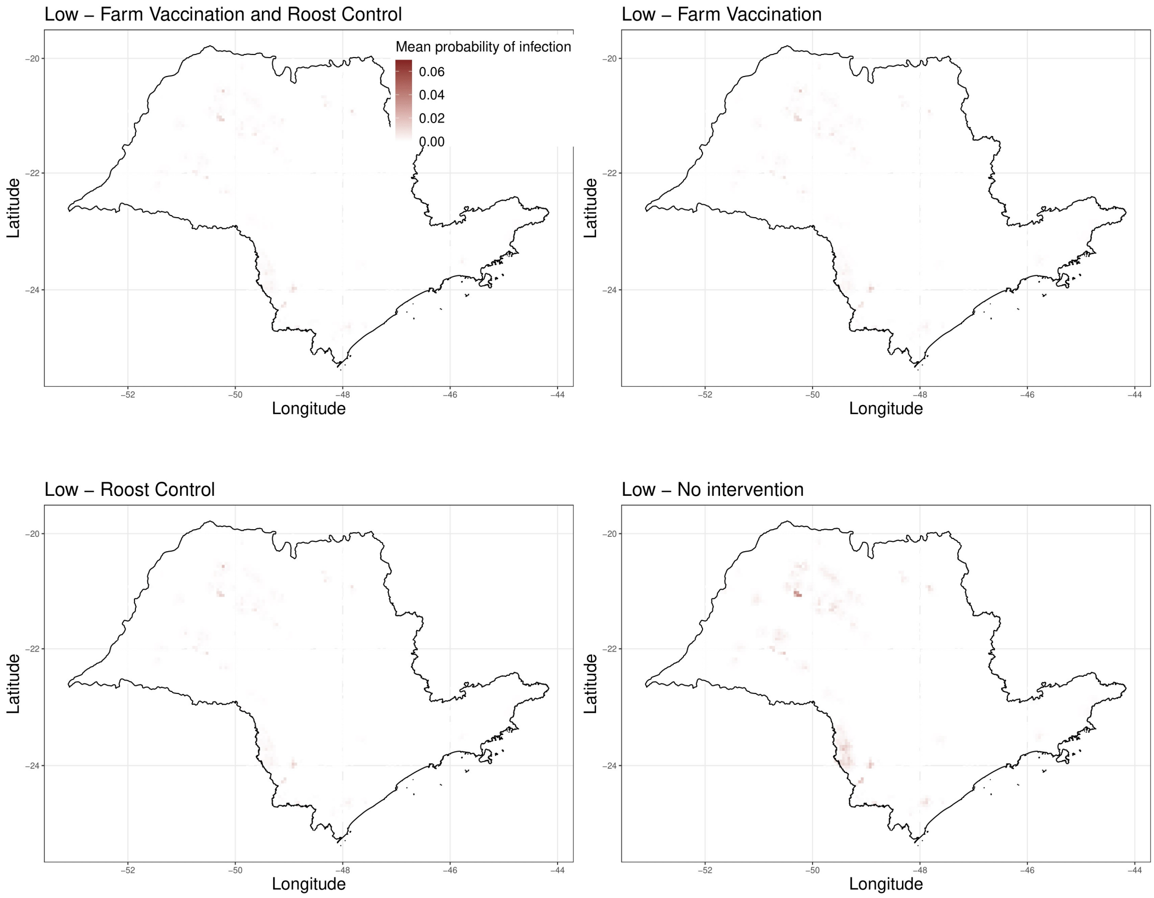

Figure S8 Spillover risk to farms measured as the probability of detected and undetected infections, among all simulations with initial infection in low suitability environment, for each intervention strategy. The value per pixel shown is the average across the farms within the pixel (3’ latitude times 3’ longitude, i.e. approx. 5.5 times 5.5 km square).

Distribution of detection period comparison

Due to high uncertainty in the *lognormal* distribution parameters for the detection period in farms, a comparison was carried out to evaluate the effect of this distribution in the results. Both sets of parameters are described in the *Detection time period in farms* section. The results in the main manuscript are based on simulations with DD1. The model was re-calibrated using the values for (see Table S2); the posterior distribution of the fitting are shown in Figure S5. Subsequently, the effectiveness of intervention strategies on the number of outbreaks in farms and the geographical spread from a single infected roost in one year are compared with the main results for the most expected initial settings of high suitability, see Figure S9.

Although qualitatively they both yield similar results, when the detection period in farms is shorter on average and driven by the less dispersed distribution (DD2 compared to DD1, see Figure S1), both the number of outbreaks on farms and the geographical spread are higher without intervention. However, the effect of the interventions is stronger with DD2, with fewer outbreaks and a lower geographical dispersion across the three intervention scenarios.

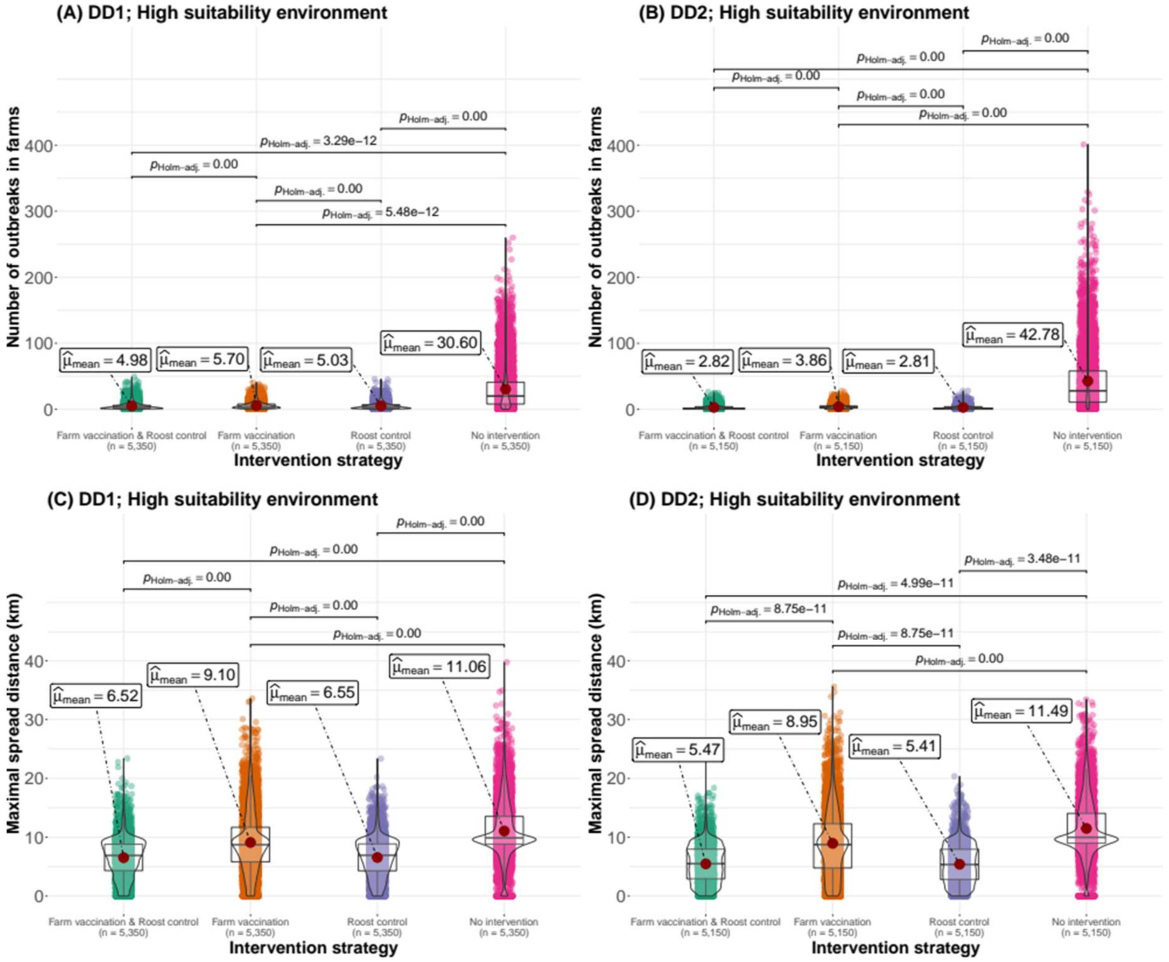

Figure S9 (Top: A-B) Distribution of the number of outbreaks (i.e. infection detections) in farms for different combinations of interventions. **(Bottom: C-D)** Distribution of maximal distances of virus spread from a single initial infection in a roost to a farm in one year in kilometers, including no virus spillovers to farms, i.e. zero distances; for different combinations of interventions in a high suitability setting.

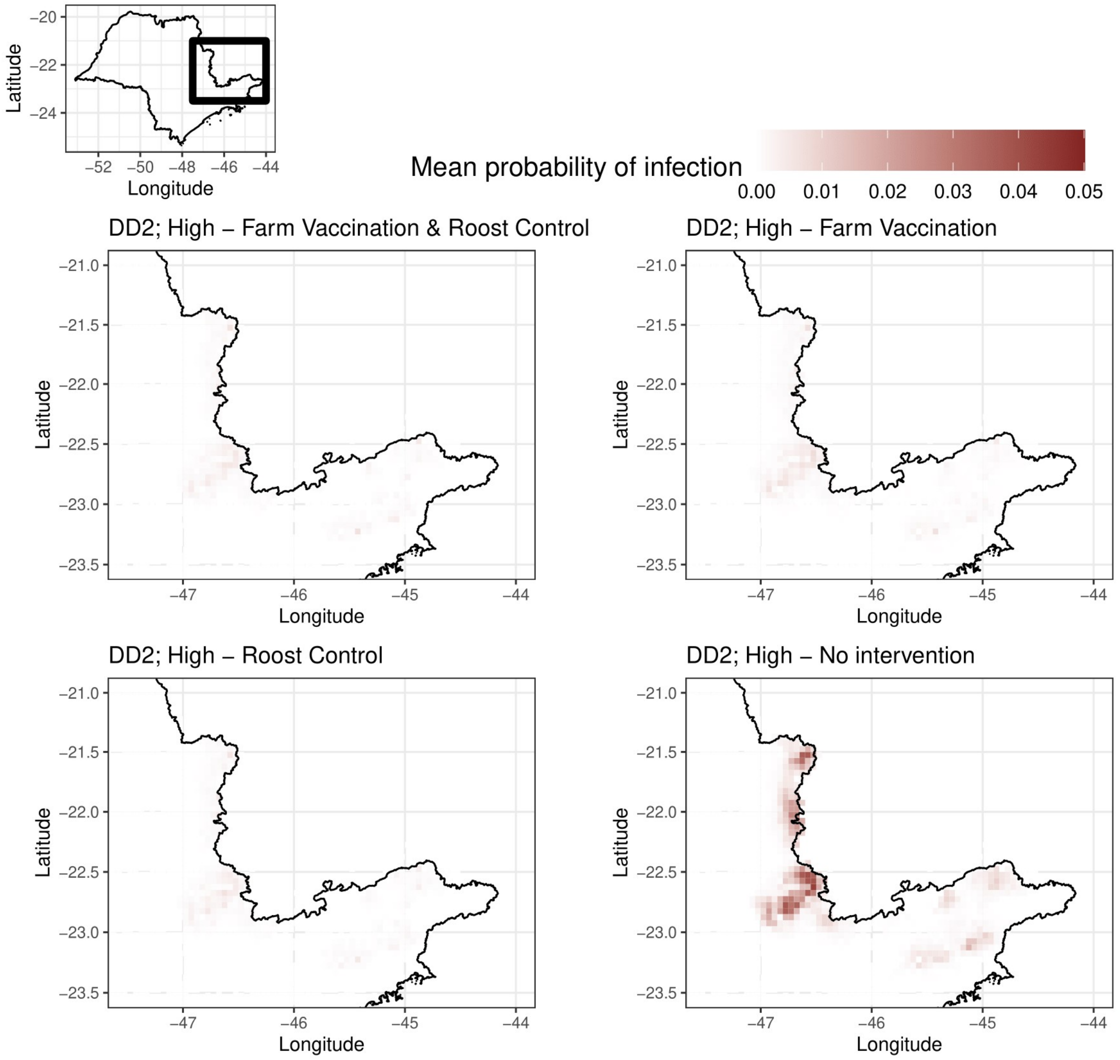

Figure S10 Spillover risk to farms measured as the probability of detected and undetected infections, among all simulations with initial infection in high suitability environment, for each intervention strategy. The value per pixel shown is the average across the farms within the pixel (square 3' latitude times 3' longitude, i.e. approx. 30 km2). This figure is akin to Figure 5 in the main text, here with DD2 instead of DD1.

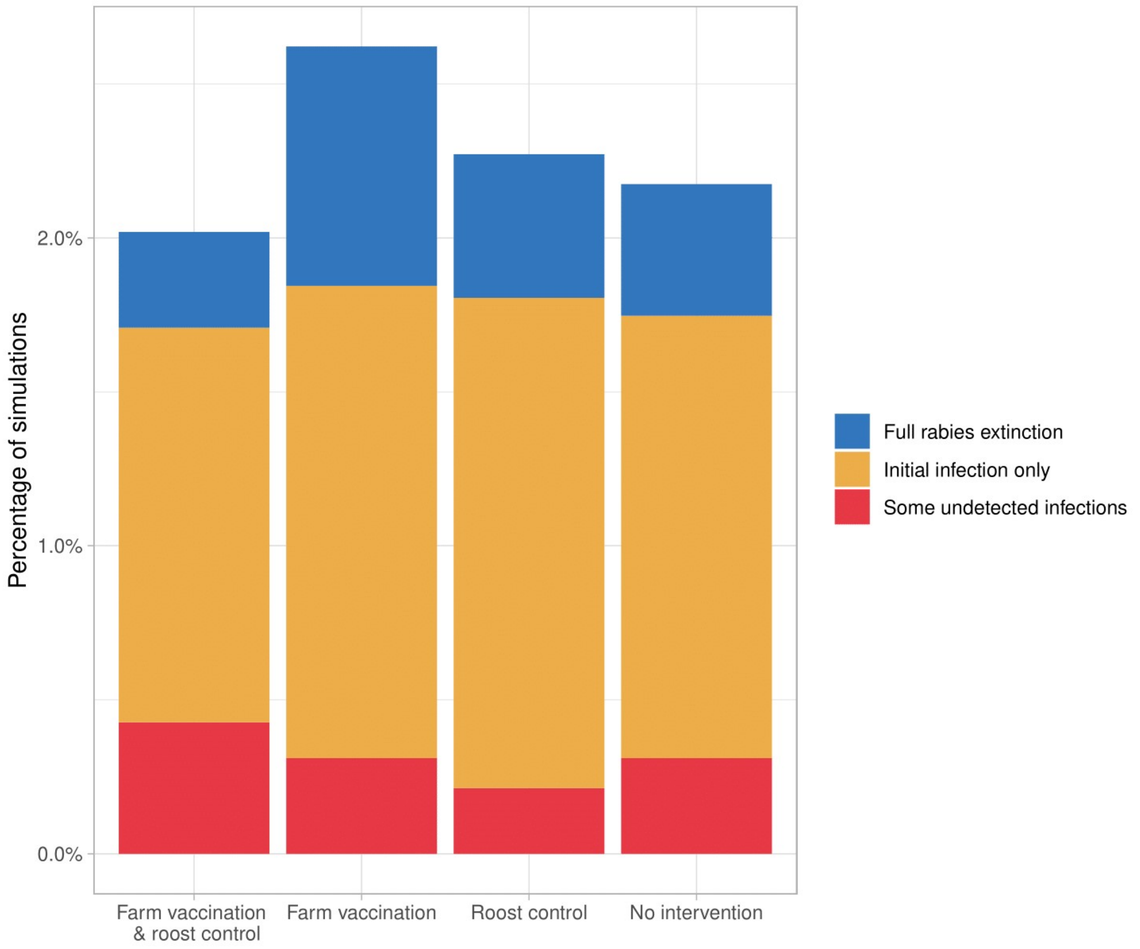

Figure S11 Percentage of runs where rabies is fully extinct **(blue)**, rabies remains only in the initial roost without spreading further **(yellow)**, and rabies had a limited spread to farms but is undetected at the end of the one-year simulation period **(red)**, in a high suitability environment for each intervention strategy. This figure is akin to Figure 6 in the main text, here with DD2 instead of DD1.

References

Anderson, R. P. (2013). A framework for using niche models to estimate impacts of climate change on species distributions. *Annals of the New York Academy of Sciences*, *1297*, 8–28. https://doi.org/10.1111/nyas.12264

Barrios, J. M., Verstraeten, W. W., Maes, P., Aerts, J.-M., Farifteh, J., & Coppin, P. (2012). Using the Gravity Model to Estimate the Spatial Spread of Vector-Borne Diseases. *International Journal of Environmental Research and Public Health*, *9*(12), 4346–4364. https://doi.org/10.3390/ijerph9124346

Beaumont, M. A., Zhang, W., & Balding, D. J. (2002). Approximate Bayesian Computation in Population Genetics. *Genetics*, *162*(4), 2025–2035. https://doi.org/10.1093/genetics/162.4.2025

Becker, D. J., Broos, A., Bergner, L. M., Meza, D. K., Simmons, N. B., Fenton, M. B., Altizer, S., & Streicker, D. G. (2020). Temporal patterns of vampire bat rabies and host connectivity in Belize. *Transboundary and Emerging Diseases*, *00*, 1–10. https://doi.org/10.1111/tbed.13754

Benavides, J. A., Valderrama, W., & Streicker, D. G. (2016). Spatial expansions and travelling waves of rabies in vampire bats. *Proceedings of the Royal Society B: Biological Sciences*, *283*(1832), 20160328. https://doi.org/10.1098/rspb.2016.0328

Chatterjee, S., & Hadi, A. S. (2006). *Regression Analysis by Example* (4th ed.). John Wiley & Sons.

Colombi, D., Poletto, C., Nakouné, E., Bourhy, H., & Colizza, V. (2020). Long-range movements coupled with heterogeneous incubation period sustain dog rabies at the

national scale in Africa. *PLOS Neglected Tropical Diseases*, *14*(5), e0008317. https://doi.org/10.1371/journal.pntd.0008317

Delpietro, H. A., Russo, R. G., Carter, G. G., Lord, R. D., & Delpietro, G. L. (2017). Reproductive seasonality, sex ratio and philopatry in Argentina's common vampire bats. *Royal Society Open Science*, *4*, 160959. https://doi.org/10.1098/rsos.160959

Escobar, L. E., Peterson, A. T., Papeş, M., Favi, M., Yung, V., Restif, O., Qiao, H., & Medina-Vogel, G. (2015). Ecological approaches in veterinary epidemiology: Mapping the risk of bat-borne rabies using vegetation indices and night-time light satellite imagery. *Veterinary Research*, *46*, 92. https://doi.org/10.1186/s13567-015-0235-7

Garg, S. R. (2014). Rabies Manifestations and Diagnosis. In S. R. Garg, *Rabies in Man and Animals* (1st ed., pp. 37–49). Springer India. https://doi.org/10.1007/978-81-322-1605-6_4

Giménez, A. L., Giannini, N. P., Schiaffini, M. I., & Martin, G. M. (2015). Geographic and potential distribution of a poorly known South American bat, *Histiotus macrotus* (Chiroptera: Vespertilionidae). *Acta Chiropterologica*, *17*(1), 143–158. https://doi.org/10.3161/15081109ACC2015.17.1.012

Gylys, L., Chomel, B. B., & Gardner, I. A. (1998). Epidemiological surveillance of rabies in Lithuania from 1986 to 1996. *Revue Scientifique et Technique de l'OIE*, *17*(3), 691–698. https://doi.org/10.20506/rst.17.3.1129

Hahn, M. B., Epstein, J. H., Gurley, E. S., Islam, M. S., Luby, S. P., Daszak, P., & Patz, J. A. (2014). Roosting behaviour and habitat selection of *Pteropus giganteus* reveal

potential links to Nipah virus epidemiology. *Journal of Applied Ecology*, *51*(2), 376–387. https://doi.org/10.1111/1365-2664.12212

Hayes, M. A., & Piaggio, A. J. (2018). Assessing the potential impacts of a changing climate on the distribution of a rabies virus vector. *PLOS ONE*, *13*(2), e0192887. https://doi.org/10.1371/journal.pone.0192887

Hijmans, R. J. (2021). *geosphere: Spherical Trigonometry* [R package version 1.5-14]. https://CRAN.R-project.org/package=geosphere

Kunz, T. H., & Fenton, M. B. (2003). *Bat Ecology*. University of Chicago Press.

Lee, D. N., Papeş, M., & Bussche, R. A. V. D. (2012). Present and Potential Future Distribution of Common Vampire Bats in the Americas and the Associated Risk to Cattle. *PLOS ONE*, *7*(8), e42466. https://doi.org/10.1371/journal.pone.0042466

Lopes, J. S., & Beaumont, M. A. (2010). ABC: A useful Bayesian tool for the analysis of population data. *Infection, Genetics and Evolution*, *10*(6), 825–832. https://doi.org/10.1016/j.meegid.2009.10.010

Maher, S. P., Kramer, A. M., Pulliam, J. T., Zokan, M. A., Bowden, S. E., Barton, H. D., Magori, K., & Drake, J. M. (2012). Spread of white-nose syndrome on a network regulated by geography and climate. *Nature Communications*, *3*, 1306. https://doi.org/10.1038/ncomms2301

Mollentze, N., Streicker, D. G., Murcia, P. R., Hampson, K., & Biek, R. (2020). Virulence mismatches in index hosts shape the outcomes of cross-species transmission. *Proceedings of the National Academy of Sciences*, *117*(46), 28859–28866. https://doi.org/10.1073/pnas.2006778117

Naimi, B., & Araújo, M. B. (2016). sdm: A reproducible and extensible R platform for species distribution modelling. *Ecography*, *39*(4), 368–375. https://doi.org/10.1111/ecog.01881

Nicolas, G., Apolloni, A., Coste, C., Wint, G. R. W., Lancelot, R., & Gilbert, M. (2018). Predictive gravity models of livestock mobility in Mauritania: The effects of supply, demand and cultural factors. *PLOS ONE*, *13*(7), e0199547. https://doi.org/10.1371/journal.pone.0199547

Nishiura, H. (2007). Early efforts in modeling the incubation period of infectious diseases with an acute course of illness. *Emerging Themes in Epidemiology*, *4*, 2. https://doi.org/10.1186/1742-7622-4-2

Owens, H. L., Campbell, L. P., Dornak, L. L., Saupe, E. E., Barve, N., Soberón, J., Ingenloff, K., Lira-Noriega, A., Hensz, C. M., Myers, C. E., & Peterson, A. T. (2013). Constraints on interpretation of ecological niche models by limited environmental ranges on calibration areas. *Ecological Modelling*, *263*, 10–18. https://doi.org/10.1016/j.ecolmodel.2013.04.011

Phillips, S. J., Anderson, R. P., & Schapire, R. E. (2006). Maximum entropy modeling of species geographic distributions. *Ecological Modelling*, *190*(3), 231–259. https://doi.org/10.1016/j.ecolmodel.2005.03.026

Phillips, S. J., & Dudík, M. (2008). Modeling of species distributions with Maxent: New extensions and a comprehensive evaluation. *Ecography*, *31*(2), 161–175. https://doi.org/10.1111/j.0906-7590.2008.5203.x

Prada Jiménez de Cisneros, J., Stear, M. J., Mair, C., Singleton, D., Stefan, T., Stear, A., Marion, G., & Matthews, L. (2014). An explicit immunogenetic model of gastrointestinal nematode infection in sheep. *Journal of The Royal Society Interface*, *11*(99), 20140416. https://doi.org/10.1098/rsif.2014.0416

Razgour, O., Forester, B., Taggart, J. B., Bekaert, M., Juste, J., Ibáñez, C., Puechmaille, S. J., Novella-Fernandez, R., Alberdi, A., & Manel, S. (2019). Considering adaptive genetic variation in climate change vulnerability assessment reduces species range loss projections. *Proceedings of the National Academy of Sciences*, *116*(21), 10418–10423. https://doi.org/10.1073/pnas.1820663116

Rocha, F., & Dias, R. A. (2020). The common vampire bat *Desmodus rotundus* (Chiroptera: Phyllostomidae) and the transmission of the rabies virus to livestock: A contact network approach and recommendations for surveillance and control. *Preventive Veterinary Medicine*, *174*, 104809. https://doi.org/10.1016/j.prevetmed.2019.104809

Rocha, F., Ulloa-Stanojlovic, F. M., Rabaquim, V. C. V., Fadil, P., Pompei, J. C., Brandão, P. E., & Dias, R. A. (2020). Relations between topography, feeding sites, and foraging behavior of the vampire bat, *Desmodus rotundus*. *Journal of Mammalogy*, *101*(1), 164–171. https://doi.org/10.1093/jmammal/gyz177

Sartwell, P. E. (1966). The incubation period and the dynamics of infectious disease. *American Journal of Epidemiology*, *83*(2), 204–216. https://doi.org/10.1093/oxfordjournals.aje.a120576

Soberón, J., & Peterson, A. T. (2005). Interpretation of Models of Fundamental Ecological Niches and Species' Distributional Areas. *Biodiversity Informatics*, *2*, 1–10. https://doi.org/10.17161/bi.v2i0.4

Streicker, D. G., Winternitz, J. C., Satterfield, D. A., Condori-Condori, R. E., Broos, A., Tello, C., Recuenco, S., Velasco-Villa, A., Altizer, S., & Valderrama, W. (2016). Host–pathogen evolutionary signatures reveal dynamics and future invasions of vampire bat rabies. *Proceedings of the National Academy of Sciences*, *113*(39), 10926–10931. https://doi.org/10.1073/pnas.1606587113

Tojinbara, K., Sugiura, K., Yamada, A., Kakitani, I., Kwan, N. C. L., & Sugiura, K. (2016). Estimating the probability distribution of the incubation period for rabies using data from the 1948–1954 rabies epidemic in Tokyo. *Preventive Veterinary Medicine*, *123*, 102–105. https://doi.org/10.1016/j.prevetmed.2015.11.018

Zeale, M., & Carr, A. (2019). *An examination of roost site selection by barbastelle bats in the Bovey valley using fine-scale ecological niche modelling* (pp. 1–13) [Technical report]. Woodland Trust.
